# medicalcoder: a unified and longitudinally aware framework for International Classification of Diseases code-based comorbidity assessment in R

**DOI:** 10.1093/jamiaopen/ooag182

**Published:** 2026-09-24

**Authors:** Peter E DeWitt, Seth Russell, James A Feinstein, Margaret N Rebull, Tellen D Bennett

**Affiliations:** Department of Biomedical Informatics, University of Colorado School of Medicine, University of Colorado Anschutz Medical Campus, Aurora, CO 80045, United States; Department of Biomedical Informatics, University of Colorado School of Medicine, University of Colorado Anschutz Medical Campus, Aurora, CO 80045, United States; Adult and Child Center for Outcomes Research and Delivery Science, University of Colorado Anschutz Medical Campus, Aurora, CO 80045, United States; Children’s Hospital Colorado, Aurora, CO 80045, United States; Department of Pediatrics, University of Colorado Anschutz Medical Campus, Aurora, CO 80045, United States; Department of Biomedical Informatics, University of Colorado School of Medicine, University of Colorado Anschutz Medical Campus, Aurora, CO 80045, United States; Department of Biomedical Informatics, University of Colorado School of Medicine, University of Colorado Anschutz Medical Campus, Aurora, CO 80045, United States; Section of Critical Care Medicine, Department of Pediatrics, University of Colorado School of Medicine, University of Colorado Anschutz Medical Campus, Aurora, CO 80045, United States

**Keywords:** comorbidity, International Classification of Diseases (ICD), software, longitudinal studies, pediatrics

## Abstract

**Objectives:**

Comorbidity algorithms derived from International Classification of Diseases (ICD) codes are central to risk adjustment and cohort characterization, but existing software inconsistently handles mixed ICD-9/10 data and typically operates at the encounter level. We developed *medicalcoder* to address these limitations.

**Materials and Methods:**

*medicalcoder* is an R package with an internal ICD database and unified interface for applying variants of the Charlson, Elixhauser, and Pediatric Complex Chronic Conditions (PCCC) algorithms. It supports full and compact ICD codes, mixed ICD versions, present-on-admission (POA) and primary diagnosis indicators, and encounter-level and cumulative longitudinal flagging.

**Results:**

The *medicalcoder* package requires only R ≥ 3.5.0; no additional packages, internet access, or external data are required. Outputs are consistent with published reference implementations. Longitudinal flagging identifies additional comorbidity flags under a carry-forward assumption while accounting for POA or technology-dependent logic.

**Conclusions:**

*medicalcoder* provides a unified, reproducible framework for ICD-based comorbidity assessment in modern clinical datasets.

## Background and significance

Accurate identification of clinical conditions in large healthcare datasets depends on ICD codes.^1–8^ Comorbidity algorithms built on these codes support risk adjustment, cohort characterization, and reproducible studies of patient populations.^1^^,^^9–11^

Existing software for flagging comorbidities typically implements 1 or 2 algorithms, most often variants of Charlson and Elixhauser, supports either ICD-9 or ICD-10 (but not both simultaneously), and requires manual reconciliation of mixed-version datasets.^4^^,^^12–16^

Other R packages for working with ICD codes and comorbidities include the archived *icd* package, which provides ICD validation, manipulation, and comorbidity mapping,^17^ and the *comorbidity* and *pccc* packages, which implement specific algorithms.^12^^,^^13^

Despite the importance of longitudinal data in medical research,^18–20^ comorbidity software typically operates at the encounter level.^5^^,^^12–16^^,^^21–24^ While the Agency for Healthcare Research and Quality (AHRQ)^5^ Elixhauser variants account for POA indicators and exemptions, they do not propagate conditions across encounters within a patient record. As a result, conditions that arise during acute care and are excluded from the index encounter may be missed in subsequent encounters unless the relevant ICD codes are re-reported and flagged as POA.

This limitation is particularly important given the high prevalence of chronic disease in the United States, where 76.4% of adults report at least 1 chronic condition and 59.5% report 2 or more.^25^ Failing to account for longitudinal patient history can therefore lead to systematic under-ascertainment of comorbidities and misclassification of patient risk.

We developed *medicalcoder* as a unified, POA-aware application programming interface supporting multiple algorithms, algorithm-specific requirements, mixed ICD versions and types, and longitudinal data.

## Objective

Develop a self-contained R package with an internal ICD database capable of applying multiple comorbidity algorithms to clinical datasets without external dependencies, internet access, or data resources. The package should support simultaneous ICD-9 and ICD-10 coding, accept full and compact ICD codes, incorporate POA and primary diagnosis indicators, and enable longitudinal comorbidity assessment across patient encounters.

## Methods

*medicalcoder* was designed to run within restricted computing environments. No namespaces are imported, and no network access is required. R ≥ 3.5.0 is the only required installation and runtime dependency.

As of *medicalcoder* version 0.9.0, the internal ICD database includes ICD-9 diagnostic codes from the Centers for Disease Control and Prevention (CDC) and ICD-9-CM (Clinical Modification) and ICD-9-PCS codes from the Centers for Medicare and Medicaid Services (CMS). ICD-10 diagnostic codes from the CDC and the World Health Organization (WHO) are included, along with ICD-10-CM and ICD-10-PCS codes from CMS, ICD-10-AM (Australian Modification) data from the Independent Health and Aged Care Pricing Authority, and ICD-10-SE (Swedish modification) data from Socialstyrelsen. Future releases will incorporate published updates to supported code sets and additional national modifications.

Charlson variants include Deyo (as denoted in Quan et al.^1^), Quan et al.,^1^^,^^2^ Glasheen et al.,^6^ Ludvigsson et al.,^7^^,^^8^ Sundararajan et al.,^26^ and Beyrer.^27^ Elixhauser variants include the original Elixhauser et al.,^28^ AHRQ Web (ICD-9 based as denoted in Quan et al.^1^), Quan et al.,^1^ and AHRQ ICD-10 fiscal-year implementations (2022-2026).^5^ Pediatric Complex Chronic Conditions variants include versions 2.0,^22^ 2.1, 3.0,^3^ and 3.1. The reference data and comorbidity mappings are versioned within each *medicalcoder* release.

*medicalcoder* maps ICD codes to comorbidity conditions using method-specific lookup tables derived from, but distinct from, its broader internal ICD database. Each table defines the ICD versions (9 or 10), code types (diagnosis or procedure), full and compact code forms, and condition mappings supported by the selected algorithm. Unlike implementations based primarily on partial string matching or regular expressions,^12^^,^^13^^,^^24^  *medicalcoder* defaults to matching input against precomputed valid ICD codes through database-style joins. For supported methods, users may select method-specific regular-expression mappings. Dataset- or row-level metadata can identify ICD version and code type, allowing mixed ICD-9 and ICD-10 diagnosis and procedure codes within a dataset or encounter. Matches are reduced to unique encounter–condition combinations before binary indicators are assigned.

Because code text alone cannot reliably distinguish jurisdictional variants such as WHO ICD-10 and ICD-10-CM, users must select a method appropriate to their source coding system and provide accurate code-system metadata.

A comprehensive test suite runs through GitHub Actions and the Comprehensive R Archive Network (CRAN), with additional compatibility testing across supported R versions. Encounter-level outputs are compared with published R, SAS, and Structured Query Language (SQL) implementations: PCCC v2.0 with *pccc*,^13^ AHRQ Elixhauser variants with the published AHRQ SAS programs,^5^ and Charlson variants with *comorbidity*^12^ and Medical Information Mart for Intensive Care IV (MIMIC-IV) Code.^24^ Testing workflows and comparisons are provided in the package repository and supplement.

We use the MIMIC-IV v3.1 dataset ^29–31^ for examples, validation, and benchmarking, not clinical inference. Pediatric comorbidity algorithms are demonstrated using adult data. We compare *medicalcoder* with MIMIC-IV code,^23^^,^^24^  *comorbidity*,^12^ and *pccc.*^13^

## Results

**Figure 1.**
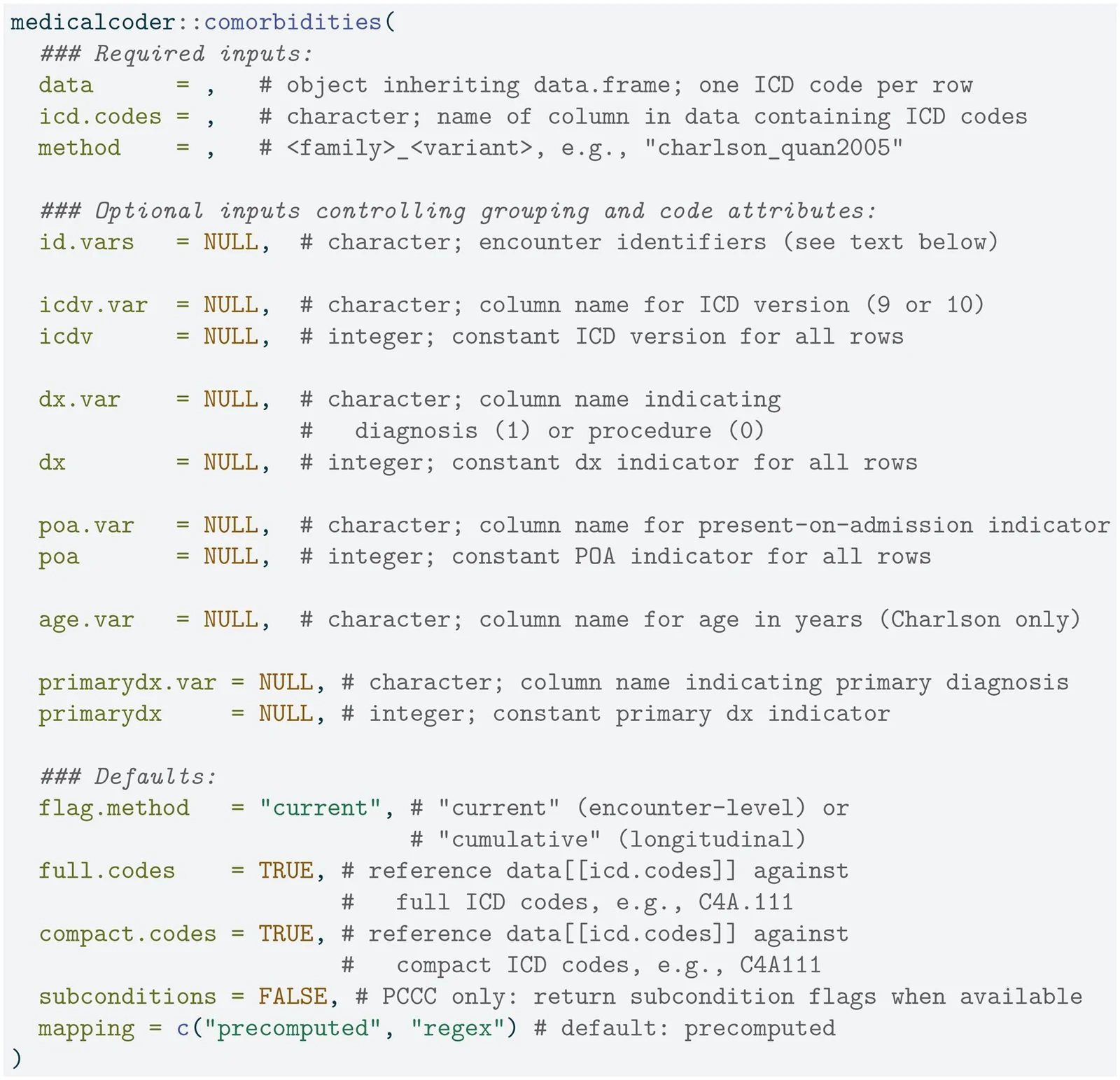
Interface for the medicalcoder::comorbidities() function. The function requires a data object containing 1 ICD code per row, the column containing ICD codes, and the comorbidity algorithm specification (eg, Charlson or Elixhauser variants). Optional arguments allow users to specify encounter identifiers, ICD version, diagnosis type, POA indicators, patient age, and primary diagnosis flags. Defaults control how comorbidities are identified, including encounter-level (flag.method = “current”) or longitudinal (flag.method = “cumulative”) evaluation and how ICD codes are matched.

The MIMIC-IV v3.1 dataset includes 7 365 770 rows of data for 364 627 unique subjects and a total of 687 203 unique hospital encounters. Records of 30 921 subjects contain both ICD-9 and ICD-10 codes, and 11 encounters contain both versions.

medicalcoder::comorbidities() can apply 21 comorbidity algorithms to long-format, 1 ICD code per row, data. Optional columns identify patients and/or encounters, ICD version and type, POA, and primary diagnosis (Figure 1). A single patient record may contain mixed ICD code versions and types in full or compact forms.

Paired arguments (eg, dx and dx.var) specify dataset-level values or row-level columns, respectively, avoiding constant-valued columns while supporting row-level variation. If both are supplied, the .var argument takes precedence and a warning is issued.

id.vars defines encounter-level summaries: with flag.method = “current”, omission treats the dataset as 1 encounter; with flag.method = “cumulative”, id.vars must include at least 2 variables defining the patient and within-patient encounter order (Section S5.1).

Output preserves the input data.frame class and includes id.vars, 1 indicator column per comorbidity, and summary columns num_cmrb and cmrb_flag. An S3 summary() method generates count and percentage tables (Table 1).

**Table 1.** Counts and percentages of encounters within the MIMIC-IV dataset flagged with Charlson comorbidities via medicalcoder::comorbidities() with method = “charlson_quan2011”.

	Encounters	%
**Condition**		
** AIDS/HIV**	3734	0.54
** Any malignancy**	47 461	6.91
** Cerebrovascular disease**	37 281	5.43
** Chronic pulmonary disease**	100 402	14.61
** Congestive heart failure**	84 715	12.33
** Dementia**	16 413	2.39
** Diabetes with chronic complications**	48 245	7.02
** Diabetes without chronic complications**	84 632	12.32
**Hemiplegia or paraplegia**	9591	1.40
** Liver disease, mild**	30 359	4.42
** Liver disease, moderate to severe**	14 071	2.05
** Metastatic solid tumor**	27 943	4.07
** Myocardial infarction**	44 702	6.50
** Peptic ulcer disease**	7165	1.04
** Peripheral vascular disease**	38 121	5.55
** Renal disease**	86 101	12.53
** Rheumatic disease**	15 858	2.31
**Total Conditions**		
**≥ 1**	347 600	50.58
**≥ 2**	194 034	28.24
**≥ 3**	96 641	14.06
**≥ 4**	40 807	5.94
**≥ 5**	13 671	1.99
**≥ 6**	3394	0.49
**≥ 7**	567	0.08
**≥ 8**	76	0.01
**≥ 9**	4	0.00

Encounter-level flagging assumes relevant ICD codes are reported, and when required, marked POA at each encounter. Omissions may under-ascertain conditions or severity.

**Figure 2.**
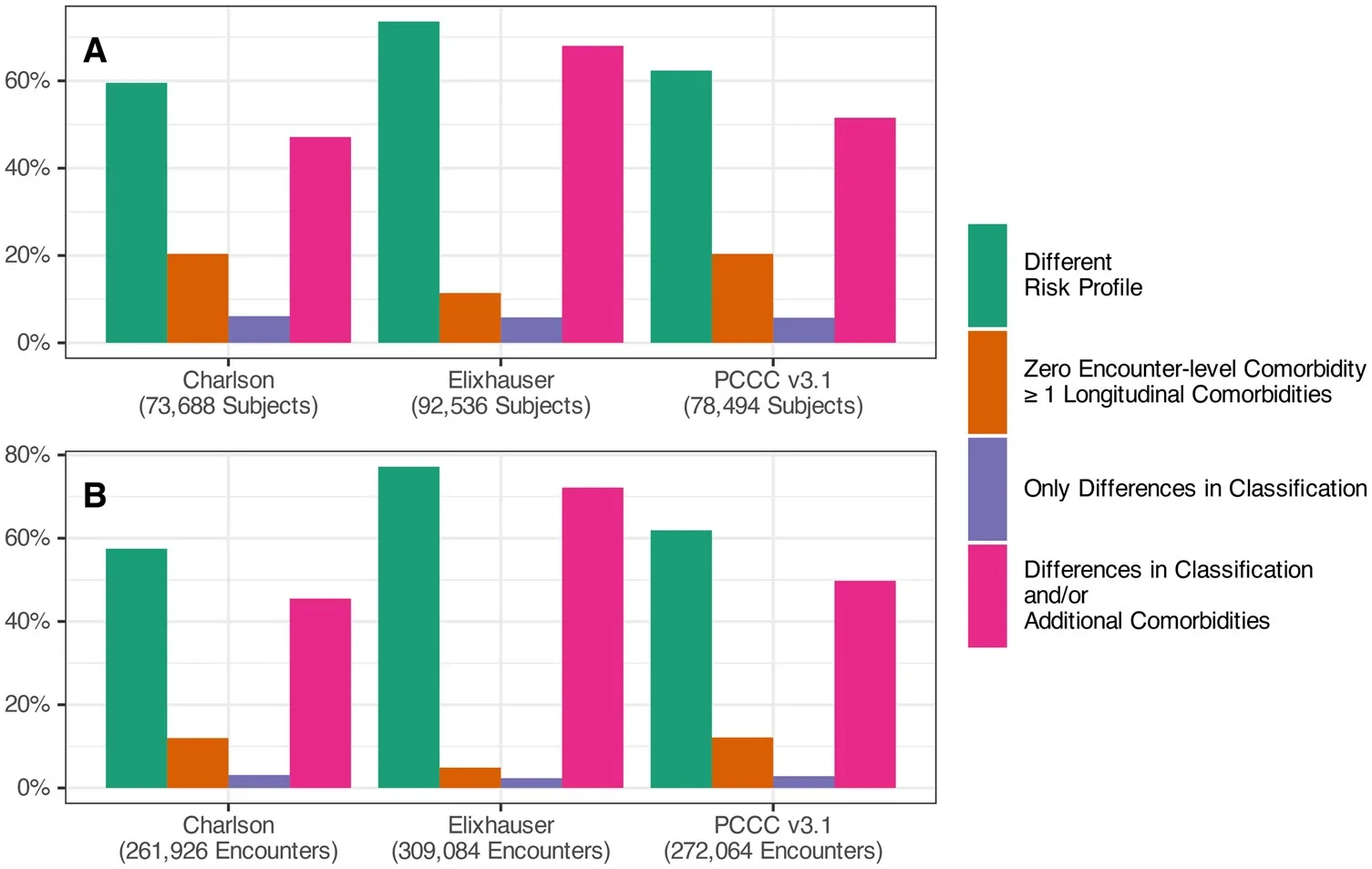
Percent of subjects and encounters in the MIMIC-IV dataset with different comorbidity profiles under flag.method = “cumulative” versus flag.method = “current”. The bars are not mutually exclusive. “Different risk profile” is an aggregation measure indicating any difference in the comorbidity classification or count between cumulative and current flagging. The remaining bars describe selected components of that aggregate, including encounters with no encounter-level comorbidity but at least 1 cumulative comorbidity, classification or severity differences with the same comorbidity count, and differences involving additional comorbidities. Panel (A) uses subjects with at least 2 encounters and at least 1 non-initial encounter with a comorbidity flagged under flag.method = “cumulative” as the denominator. Panel (B) uses non-initial encounters with at least 1 comorbidity flagged under flag.method = “cumulative” as the denominator.

Figure 2 compares cumulative and current profiles using descriptive, nonexclusive measures. “Different risk profile” denotes any difference in condition classification or count. Denominators are subjects with at least 2 encounters and a cumulative flag after the index encounter, and non-index encounters with at least 1 cumulative flag. Among 92 536 eligible Elixhauser subjects, 68 085 (73.6%) had a different profile on at least 1 encounter, and 10 553 (11.4%) had no current but at least 1 cumulative comorbidity.

Pediatric Complex Chronic Conditions v3 and the 2022-2026 AHRQ Elixhauser algorithms require additional logic. Pediatric Complex Chronic Conditions v3 conditions depend on whether technology-dependent codes co-occur (or eventually co-occur) with non–technology-dependent codes (Table 2). For AHRQ Elixhauser variants, POA-required conditions are flagged when POA is present, or the ICD code is POA-exempt (Section S8.4).

**Table 2.** Flagging of comorbidities under PCCC v3 for MIMIC-IV subject 10728333.

	Metabolic		Miscellaneous		Respiratory		Any Tech		Comorbidity Count	
Encounter	Current	Cumulative	Current	Cumulative	Current	Cumulative	Current	Cumulative	Current	Cumulative
**1**					^a^	^a^			0	0
**2**						^b^			0	0
**3**			^a^	^a^		^b^			0	0
**4**	DxPr	DxPr		Tech		Tech		1	1	3
**5**	DxPr	DxPr		Tech		Tech		1	1	3
**6**	DxPr	DxPr		Tech	DxPr	DxPrTech		1	2	3
**7**	DxPr	DxPr		Tech		DxPrTech		1	1	3
**8**	DxPr	DxPr		Tech		DxPrTech		1	1	3
**9**	DxPr	DxPr		Tech		DxPrTech		1	1	3
**10**	DxPr	DxPr		Tech		DxPrTech		1	1	3
**11**		DxPr		Tech		DxPrTech		1	0	3
**12**		DxPr		Tech		DxPrTech		1	0	3
**13**	DxPr	DxPr		Tech	Tech	DxPrTech	1	1	2	3

Cells marked with DxPr denote that the comorbidity was flagged due to an ICD code that is not technology-dependent. Tech denotes flagging of a comorbidity based on the presence of at least 1 technology-dependent ICD code and the presence of at least 1 non–technology-dependent code for another comorbidity. DxPrTech denotes flagging due to both technology-dependent and non–technology-dependent ICD codes. Under the Any Tech columns, we mark the encounters where some tech dependence is identified. For this patient, on encounter 1, a technology-dependent ICD code for a respiratory comorbidity was found in the subject’s record. Because no non–technology-dependent code was observed on encounter 1, under PCCC v3 this subject has no comorbidities for encounter 1. This persists for encounters 2 and 3 with respect to respiratory, and on encounter 3 for miscellaneous as well. On encounter 4, a non–technology-dependent code for a metabolic comorbidity was reported. Under a cumulative flagging paradigm, the miscellaneous and respiratory comorbidities are also flagged as the reported codes from encounters 3 and 1 have been carried forward, respectively. Under the “current” flagging method, miscellaneous is never flagged as the technology-dependent code never occurs on the same encounter as the non–technology-dependent code.

a A technology-dependent code was reported on this encounter. Since no non–technology-dependent code was reported, no comorbidities are flagged.

b A technology-dependent code has been carried forward in the record. Since no non–technology-dependent code was reported on this, or a prior, encounter, no comorbidities are flagged.

Across encounter-level comparisons with MIMIC-IV code and *comorbidity*, *medicalcoder* showed 100% agreement for shared condition flags after harmonizing output conventions; PCCC v2.0 discordance with *pccc* was limited to documented technology-dependence and transplant-category differences (Table S3).

The comparators all use some form of partial string matching for mapping ICD codes to conditions. *medicalcoder* uses a set of precomputed valid ICD codes to map between input data and conditions using database-style joins. As a result, comparator implementations that rely on prefix or regular-expression matching can return false-positive flags when an invalid input string starts with a valid ICD code substring. For example, the ICD-10 code C189 maps to “any malignancy” under Charlson; *comorbidity* maps C189NOTACODE to that condition because the string starts with C18. *medicalcoder*, by default, does not map the invalid string to a condition.

**Figure 3.**
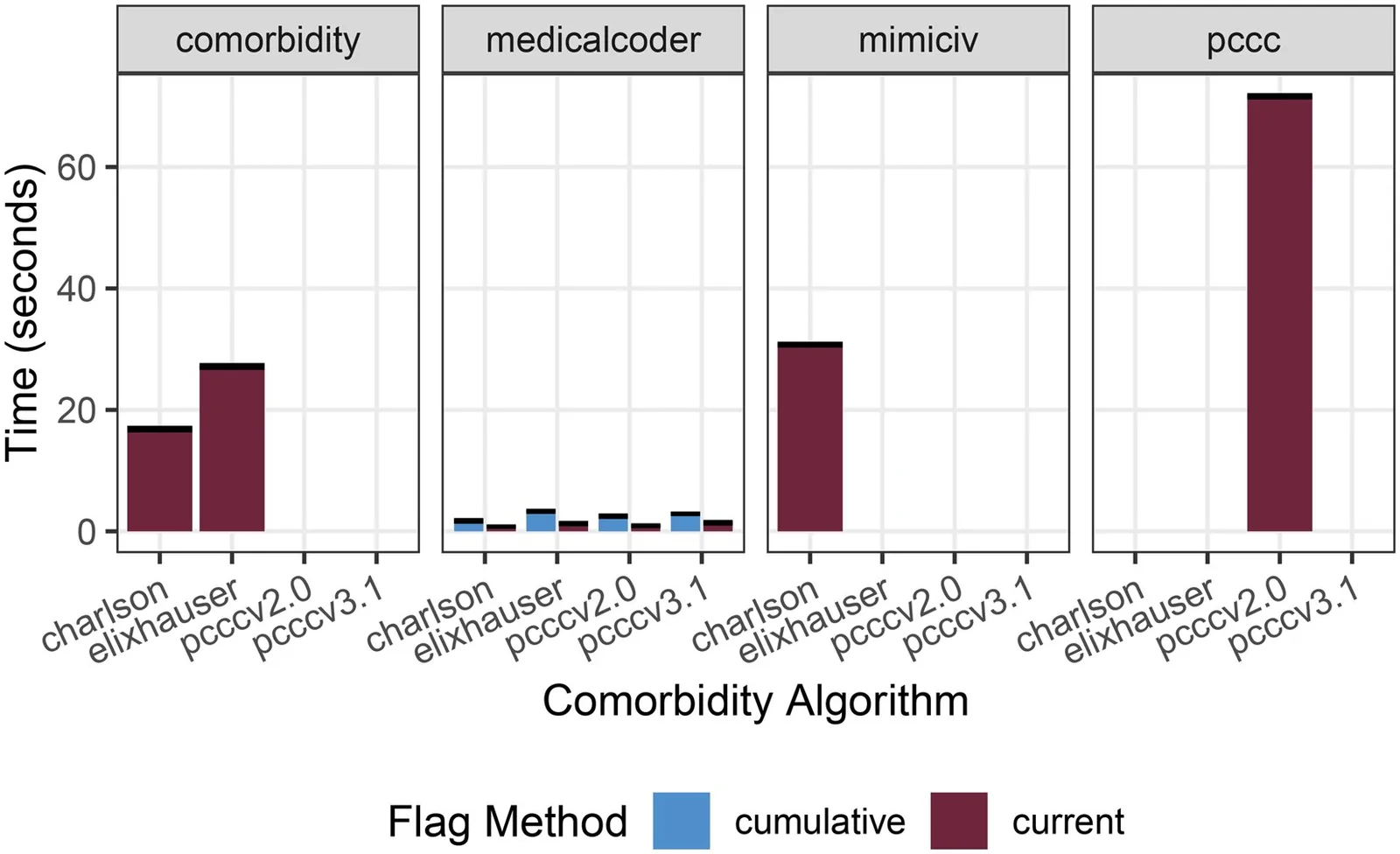
The median time (in seconds; bar height), and IQR (error bars), required to flag comorbidities in the MIMIC-IV v3.1 dataset (7 365 770 rows, 364 627 unique subjects, 687 203 unique encounters) by utility (panels) and flagging method for *N* = 10 iterations. The MIMIC-IV code time is based on running RSQLite code for an in-memory database. R packages *comorbidity* and *pccc* require multiple calls and aggregations to handle the mixed use of ICD-9 and ICD-10 within the MIMIC-IV dataset. Applying each of the comorbidity algorithms and flagging methods within *medicalcoder* requires only modifying the method and flag.method argument to the medicalcoder::comorbidities() call. Some panels are sparse, because each comparator supports fewer methods. The times were recorded when building the supplement in R version 4.6.1 (2026-06-24) on macOS Tahoe 26.5.2 with an Apple M5 Pro and 48.0 GB RAM.

Precomputed ICD-to-condition maps reduce false-positive flags compared with partial string matching and reduce computational expense. Benchmarking in the supplement and Figure 3 shows that *medicalcoder* requires less time to compute comorbidity flags in the MIMIC-IV examples.

*medicalcoder* provides ICD utility functions for data preparation and validation. medicalcoder::get_icd_codes() exposes the internal ICD database, and medicalcoder::is_icd() validates candidate codes across full and compact representations and mixed ICD-9/ICD-10 datasets.

## Discussion

*medicalcoder* originated as a PCCC implementation,^3^^,^^4^ for which reproducible classification required a version-aware ICD database that subsequently supported Charlson and Elixhauser algorithms. The package imports no namespaces and can be installed wherever R ≥ 3.5.0 is available, including restricted environments. Pinning the package version locks its ICD database and comorbidity mappings, supporting reproducibility. “Dependency-free” applies to installation and runtime; tests, documentation, benchmarks, and manuscript workflows require the external tools and data described here and in the supplement.

*medicalcoder* addresses a methodological gap in existing comorbidity software: the lack of support for longitudinal comorbidity assessment. Most implementations operate strictly at the encounter level, implicitly assuming that comorbid conditions are either repeatedly coded or clinically irrelevant once absent from the current record. As demonstrated in Table 2, this assumption can lead to systematic under-ascertainment of comorbidities, particularly for chronic conditions or conditions governed by POA rules or technology-dependent classification criteria. By tracking condition history across encounters, *medicalcoder* supports comorbidity classification based on prior observed diagnoses under an explicit carry-forward assumption.

The utility of cumulative flags depends on whether every ICD code mapped to a condition represents persistent disease. For example, carry-forward of uncomplicated diabetes is defensible for the Quan et al. Charlson and Elixhauser mappings,^1^ which identify non-gestational diabetes. In the AHRQ fiscal year 2022 and beyond mappings, however, uncomplicated diabetes also includes ICD-10 codes under the O24 category for gestational diabetes, a pregnancy-related and potentially transient condition. Because *medicalcoder* carries forward the comorbidity flag rather than the originating ICD code, subsequent output cannot distinguish gestational from non-gestational diabetes. Thus, cumulative uncomplicated diabetes flags may be reasonable with Quan-style mappings but require additional qualification with AHRQ-style mappings.

Malignancy illustrates a related limitation. Suppose C18.2, “malignant neoplasm of the colon,” is reported. Upon resolution of the neoplasm, the C18.2 code should no longer be reported, and the ICD-10 code Z85.038, “personal history of malignant neoplasm of the large intestine,” should be reported. The cumulative flag remains positive on and after the encounter with the initial C18.2 report and therefore represents current or previously observed malignancy rather than current malignancy alone. However, because Z85.038 is not included in any of the comorbidity mappings, a history-only encounter preceding any mapped active-malignancy code remains unflagged. Cumulative malignancy flags should therefore be interpreted as indicating current or previously observed malignancy, not a comprehensive current-or-history phenotype.

The magnitude and interpretation of cumulative flagging may differ in pediatric, outpatient, claims, non-ICU, and non-US datasets compared to the results shown using the MIMIC-IV v3.1 dataset because coding density, encounter spacing, POA availability, and condition persistence vary across settings.

The unified interface provided by *medicalcoder* further reduces analytic complexity. Mixed ICD-9 and ICD-10 datasets, full and compact codes, diagnostic and procedure codes, and algorithm-specific nuances are handled within a single function call. This contrasts with existing workflows that require dataset partitioning, multiple method calls, and post hoc reconciliation, each of which can introduce error and irreproducibility.

The included ICD utility functions also support exploratory and preprocessing tasks using the same reference data employed by the comorbidity algorithms. This reduces reliance on ad hoc code lists and external reference tables when defining cohorts or filtering diagnosis and procedure codes in large clinical datasets.

Testing against published SAS, SQL, and R reference implementations demonstrates that *medicalcoder* produces results consistent with established encounter-level algorithms while offering additional flexibility.

To our knowledge, no available R package jointly supports multiple comorbidity algorithms, mixed ICD versions, POA logic, and cumulative longitudinal flagging within a single, reproducible framework. *medicalcoder* has these capabilities and enables researchers to consistently characterize patient complexity and evaluate how different comorbidity definitions influence analytic results.

## Conclusions

*medicalcoder* is a self-contained R package with an internal ICD database that can apply many comorbidity algorithms while accounting for ICD version, ICD type, POA, primary diagnosis, and full and compact ICD codes. Codes can be flagged for individual encounters or longitudinally within a patient record.

## Supplementary Material

ooag182_Supplementary_Data

## Data Availability

The *medicalcoder* R package is available from the CRAN at https://cran.r-project.org/package=medicalcoder. Developmental versions, additional testing suites, benchmarking, and examples are on GitHub at https://github.com/dewittpe/medicalcoder/. The MIMIC-IV v3.1 data are available from PhysioNet at https://physionet.org/content/mimiciv/3.1/. This article was written using Quarto version 1.9.38 (https://quarto.org/) and R version 4.6.1 (2026-06-24). All R code, materials, and dependencies can be found at https://github.com/dewittpe/medicalcoder-jamia-open/.
