# Supplementary material for: medicalcoder: a unified and longitudinally aware framework for International Classification of Diseases code-based comorbidity assessment in R
Source: JAMIA Open. 2026 Sep 24;9(5):ooag182. doi: 10.1093/jamiaopen/ooag182 (PMC13612241; doi:10.1093/jamiaopen/ooag182)
Supplement: Supplementary file 1 [file ooag182_supplementary_data.pdf]

MIMIC-IV Data Analysis via medicalcoder

Supplement to JAMIA Open Manuscript

Table of contents

1 Objective and Setup **3**

2 Materials and Methods **4**
- 2.1 Data . . . 4
- 2.2 Comorbidity Algorithms . . . 4
- 2.3 Alternative Utilities . . . 5

3 Prepare MIMIC-IV Data For Analysis **5**

4 Investigating ICD Codes **9**
- 4.1 Utilities . . . 9
- 4.2 ICD-Codes in MIMIC-IV Data . . . 9

5 Flagging Encounter Level Comorbidities **13**
- 5.1 `medicalcoder::comorbidities()` . . . 13
  - 5.1.1 Charlson Comorbidities . . . 15
  - 5.1.2 Elixhauser Comorbidities . . . 17
  - 5.1.3 PCCC v2.0 . . . 17
- 5.2 MIMIC-IV Code . . . 18
- 5.3 `comorbidity::comorbidity()` . . . 19
  - 5.3.1 Charlson . . . 20
  - 5.3.2 Elixhauser . . . 21
- 5.4 `pccc::ccc()` . . . 22

6 Comparing Encounter-level Flags Between Utilities **23**
- 6.1 Charlson Comorbidities . . . 23
  - 6.1.1 `medicalcoder::comorbidities()` vs `comorbidity::comorbidity()` . 23
  - 6.1.2 `medicalcoder::comorbidities()` vs MIMIC-IV Code . . . 26

6.2 Elixhauser Comorbidities . . . 29
6.2.1 `medicalcoder::comorbidities()` vs `comorbidity::comorbidity()` . . . 29
6.3 PCCC . . . 32
6.3.1 `medicalcoder::comorbidities()` vs `pccc::ccc()` . . . 32
6.4 Summary of *medicalcoder* vs Other Implementations . . . 35

7 Precomputed vs Regular Expressions **35**

8 Longitudinal Flagging **41**
8.1 Severity of Disease . . . 43
8.1.1 Elixhauser . . . 45
8.1.2 PCCC . . . 46
8.2 Diabetes - Chronic vs Acute Disease . . . 50
8.3 Metastatic Tumors . . . 55
8.4 AHRQ (2022+) Example - POA . . . 57

9 Time Required To Compute **60**

10 Summarizing `medicalcoder_comorbidities` Objects **62**

11 Session Info **65**

References **66**

List of Figures

1 Charlson comorbidities by flag.method. . . . 44
2 Elixhauser comorbidities by flag.method. . . . 46
3 PCCC v2 comorbidities by flag.method. . . . 47
4 PCCC v3 comorbidities by flag.method. . . . 48
5 Time, in seconds, to flag comorbidities by *medicalcoder*, *comorbidity*, *pccc*, and MIMIC-IV Code. . . . 61

List of Tables

1 Comorbidity methods implemented in *medicalcoder* and sorted by comorbidity family and method in alphabetical order. . . . 5
2 SHA256 checksums for files in the MIMIC-IV v3.1 data. . . . 6
3 Summary of encounter-level agreement between *medicalcoder* and comparator implementations using MIMIC-IV v3.1 data, a total of 687,203 encounters. . . . 35

4 Example impact of flagging Diabetes using encounter-level data only vs a longitudinal approach. These are the Charlson comorbidities for MIMIC-IV subject_id 10009326 using `medicalcoder::comorbidities()` with `method = "charlson_quan2011"`, `flag.method = "current"` and `flag.method = "cumulative"`. 45
5 Comparison of encounter-level and cumulative flagging of PCCC v3 conditions for MIMIC-IV subject 10728333. Cells marked with DxPr denote that the comorbidity was flagged due to an ICD code that is not technology-dependent. Tech denotes flagging of a comorbidity based on the presence of at least one technology-dependent ICD code and the presence of at least one non-technology-dependent code for another comorbidity. DxPrTech denotes flagging due to both technology-dependent and non-technology-dependent ICD codes. Under the Any Tech columns we mark the encounters where some tech dependence is identified. For this patient, on encounter 1, a technology-dependent ICD code for a respiratory comorbidity was found in the subject's record. Because no non-technology-dependent code was observed on encounter 1, under PCCC v3 this subject has no comorbidities for encounter 1. This persists for encounters 2 and 3 with respect to respiratory and on encounter 3 for miscellaneous as well. On encounter 4 a non-technology-dependent code for a metabolic comorbidity was reported. Under a cumulative flagging paradigm, the miscellaneous and respiratory comorbidities are also flagged as the reported codes from encounters 3 and 1 have been carried forward respectively. Under the 'current' flagging method miscellaneous is never flagged as the technology-dependent code never occurs on the same encounter as the non-technology-dependent code. 50
6 Time, in seconds, median and IQR, to flag comorbidities via Charlson, Elixhauser, PCCC v2, and PCCC v3 and several different utilities in the MIMIC-IV v3.1 dataset. The flagging method, current or cumulative, is reported. Each method was evaluated 10 times. 62
7 Code and resulting table for a summary of the PCCC v2 conditions flagged in the MIMIC-IV dataset. 64
8 Details on the machine that generated this document. 65
9 R packages and versions used to generate this document. 65

1 Objective and Setup

This supplement provides reproducible validation and benchmarking of `medicalcoder::comorbidities()` using MIMIC-IV v3.1 data, including algorithmic agreement with established implementations and demonstration of longitudinal flagging behavior.

2 Materials and Methods

2.1 Data

We use the MIMIC-IV v3.1 Data (Johnson et al. 2024; Johnson et al. 2023; Goldberger et al. 2000) as the foundation for these examples.

The MIMIC-IV v3.1 dataset is a large, publicly available clinical database containing de-identified health data from patients admitted to intensive care units at Beth Israel Deaconess Medical Center. It includes detailed information on diagnoses, procedures, laboratory results, medications, and hospital encounters, with ICD-9-CM and ICD-10-CM/PCS codes available for billing and comorbidity research. MIMIC-IV is widely used for method development, validation, and reproducible research in clinical informatics and outcomes research.

The MIMIC-IV v3.1 dataset can be obtained so long as you meet the requirements set by PhysioNet[1].

2.2 Comorbidity Algorithms

Within the *medicalcoder* package are implementations for several variants of the Charlson, Elixhauser, and PCCC comorbidity algorithms, (Table 1) (Beyrer et al. 2021; Quan et al. 2005, 2011; Glasheen et al. 2019; Agency for Healthcare Research and Quality (AHRQ) 2025; Feudtner et al. 2014; Feinstein et al. 2024; DeWitt et al. 2026; Feinstein et al. 2018; *Complex Chronic Conditions Version 3.0* 2024; Ludvigsson et al. 2021, 2023; Sundararajan et al. 2004).

NOTE: The MIMIC-IV data consist of only adult patients. Although PCCC was designed for pediatric populations, adult MIMIC-IV data are used here solely to demonstrate algorithmic implementation.

[1]https://physionet.org/content/mimiciv/3.1/

Table 1: Comorbidity methods implemented in *medicalcoder* and sorted by comorbidity family and method in alphabetical order.

Comorbidity Method	Notes
**Charlson**	
charlson_beyrer2021	U.S. extension of Quan et al. (2005) by Beyrer et al. (2021)
charlson_cdmf2019	ICD-9 and ICD-10 defined in Glasheen et al. (2019)
charlson_deyo1992	ICD-9 codes defined in Table 1 of Quan et al. (2005)
charlson_ludvigsson2021	ICD-10-SE variant Ludvigsson et al. (2021, 2023)
charlson_mimicivcode	MIMIC-IV Charlson SQL from MIMIC-IV Code, Johnson et al. (2018)
charlson_quan2005	ICD-9 and ICD-10 defined in Table 1 of Quan et al. (2005); index scoring as reported in Table 2 of Quan et al. (2011) 'Charlson Weight'
charlson_quan2011	ICD-9 and ICD-10 defined in Table 1 of Quan et al. (2005); index scoring as reported in Table 2 of Quan et al. (2011) 'Updated Weight'
charlson_sundararajan2004	ICD-10-AM codes variant Sundararajan et al. (2004)
**Elixhauser**	
elixhauser_elixhauser1988*	ICD-9 codes defined in Table 2 of Quan et al. (2005)
elixhauser_ahrq_web	ICD-9 codes defined in Table 2 of Quan et al. (2005)
elixhauser_quan2005	ICD-9 and ICD-10 defined in Table 2 of Quan et al. (2005)
elixhauser_ahrq2022	ICD-10 codes from AHRQ for fiscal year 2022
elixhauser_ahrq2023	ICD-10 codes from AHRQ for fiscal year 2023
elixhauser_ahrq2024	ICD-10 codes from AHRQ for fiscal year 2024
elixhauser_ahrq2025	ICD-10 codes from AHRQ for fiscal year 2025
elixhauser_ahrq2026	ICD-10 codes from AHRQ for fiscal year 2026
elixhauser_ahrq_icd10	Any ICD-10 code from all elixhauser_ahrqYYYY
**PCCC**	
pccc_v2.0	ICD-9 and ICD-10 diagnostic and procedure codes consistent with pccc::ccc()
pccc_v2.1	Extended set of pccc_v2.0 codes based on pccc::ccc() and supplements to Feudtner et al. (2014)
pccc_v3.0	ICD-9 and ICD-10 diagnostic and procedure codes consistent with SAS code from Children's Hospital Association
pccc_v3.1	Extended set of pccc_v3.0 codes based on the supplements to Feinstein et al. (2024)

* Elixhauser's paper was published in 1998, not 1988. A correction to the name will be made in a future release of the *medicalcoder* package. https://github.com/dewittpe/medicalcoder/issues/50

2.3 Alternative Utilities

We compared the results from `medicalcoder::comorbidities()` to the Charlson comorbidities flagged by the MIMIC-IV code, and the R package *comorbidity* (Gasparini 2018). For Elixhauser we will compare results between `medicalcoder::comorbidities()` and `comorbidity::comorbidity()`. Lastly, we will compare PCCC version 2 results between `medicalcoder::comorbidities()` and the R package *pccc* (DeWitt et al. 2026) call `pccc::ccc()`.

3 Prepare MIMIC-IV Data For Analysis

We have stored the path to the MIMIC-IV data as a system variable on our machines as `MIMICIVDATA`. The following code is provided to help readers verify that they are using the same version of the MIMIC-IV v3.1 data as used in this document.

```r
# verify that you are looking at version 3.1 of the MIMIC-IV data
stopifnot(
  identical(
    scan(
      file = file.path(Sys.getenv("MIMICIVDATA"), "CHANGELOG.txt"),
      what = "character", sep = "\n", quiet = TRUE)[1],
    "This is the change log for MIMIC-IV v3.1."
  )
)
```

We only need four of the MIMIC-IV hospital datasets: `admissions`, `patients`, `diagnoses_icd`, and `procedures_icd`.

We will do some light formatting before using `medicalcoder::comorbidities()` to flag comorbidities. Start by importing the `admissions`, `patients`, `diagnoses_icd`, and `procedures_icd` datasets.

Table 2 gives the SHA256 checksums for these files. Each file checksum has been verified.

Table 2: SHA256 checksums for files in the MIMIC-IV v3.1 data.

sha256sum	file
a9584ed88e9ed664a2f66f86a5cf9fd175c8bb0af50e3b6115598b19e978384e	hosp/admissions.csv.gz
47665a41b2a3ad990d6f314062d5bd6d29d467b0fd402dd94662044ec8b073d2	hosp/diagnoses_icd.csv.gz
4c7507de7ecad8c5ba7647ea4d26018e88ff6672c5fc22ab59c2a5697d687cdd	hosp/patients.csv.gz
3271397d0517131f84399b698cbb0c2f435aaab0f873a3806e13b097661596f7	hosp/procedures_icd.csv.gz

```r
mimicivdata <-
  list.files(
    path = file.path(Sys.getenv("MIMICIVDATA"), "hosp"),
    pattern = "(admissions|patients|.+_icd)\\.csv\\.gz",
    full.names = TRUE
  )
names(mimicivdata) <- sub("\\.csv\\.gz$", "", basename(mimicivdata))

mimicivdata <- lapply(mimicivdata, data.table::fread)
```

We will build an additional dataset for age (in years).

```r
mimicivdata_admissions_age <-
  mimicivdata$admissions[, list(subject_id, hadm_id, admittime)]
mimicivdata_patients_age <-
  mimicivdata$patients[, list(subject_id, anchor_age, anchor_year)]

mimicivdata$ages <-
  merge(
    x = mimicivdata_admissions_age,
    y = mimicivdata_patients_age,
    all = FALSE,
    by = "subject_id"
  )
mimicivdata$ages[
  ,
  anchor_year := as.POSIXct(paste0(anchor_year, "-01-01"), format = "%Y-%m-%d")
]
mimicivdata$ages[
```

```
  ,
  age :=
    anchor_age +
    as.numeric(difftime(admittime, anchor_year, units = "days")) / 365.25
]
```

For the calls to `medicalcoder::comorbidities()` we can have all the ICD codes in one `data.frame` (data.table (Barrett et al. 2025) will be used here).

```
mimicivdata$icd <-
  data.table::rbindlist(
    list(dx = mimicivdata$diagnoses_icd, pr = mimicivdata$procedures_icd),
    idcol = "dx",
    use.names = TRUE,
    fill = TRUE
  )

# set the diagnoses indicator to an integer as expected for input to
# medicalcoder::comorbidities()
mimicivdata$icd[, dx := as.integer(dx == "dx")]
```

We put all the parts together into one data.frame (data.table)

```
mimicivDT <-
  merge(
    x = mimicivdata$patients[, .(subject_id)],
    y = mimicivdata$ages[, .(subject_id, hadm_id, age)],
    all = TRUE,
    by = c("subject_id")
  )

mimicivDT <-
  merge(
    x = mimicivdata$icd,
    y = mimicivDT,
    all = TRUE,
    by = c("subject_id", "hadm_id")
  )

mimicivDT <-
  merge(
    x = mimicivDT,
    y = mimicivdata$admissions[, .(subject_id, hadm_id, admittime)],
    all = TRUE,
    by = c("subject_id", "hadm_id")
  )
```

We need an encounter sequence variable, the `hadm_id` are not sequential.

NOTE: `admittime` can be used in the calls to `medicalcoder::comorbidities()`, but there is additional overhead and computational expense when sorting and joining by POSIXct variable. The `enc_seq` achieves the same utility with lower computational overhead.

```
data.table::setorder(mimicivDT, subject_id, admittime)
mimicivDT[, enc_seq := cumsum(!duplicated(hadm_id)), by = .(subject_id)]
data.table::setkey(mimicivDT, subject_id, enc_seq, hadm_id)
```

medicalcoder can use multiple id variables. Other utilities such as *comorbidity* and *pccc* can only use one id variable. As such we create `cid` to use in those examples.

```
mimicivDT[, cid := paste(subject_id, hadm_id, sep = "__")]

# Sanity checks:
# expect age to always be increasing as enc_seq increase
mimicivDT[
  ,
  unique(.SD),
  .SDcols = c("subject_id", "hadm_id", "admittime", "age", "enc_seq")
  ][
  ,
  c(NA_real_, diff(age)),
  by = .(subject_id)
  ][
  ,
  all(V1 >= 0, na.rm = TRUE)
  ] |>
print() |>
stopifnot()
## [1] TRUE
```

Our final `mimicivDT` object:

```
str(mimicivDT, width = 72)
## Classes 'data.table' and 'data.frame':   7365770 obs. of  11 variables:
## $ subject_id : int 10000032 10000032 10000032 10000032 10000032
##    10000032 10000032 10000032 10000032 10000032 ...
## $ hadm_id : int 22595853 22595853 22595853 22595853 22595853 22595853
##    22595853 22595853 22595853 22841357 ...
## $ dx : int 1 1 1 1 1 1 1 1 0 1 ...
## $ seq_num : int 1 2 3 4 5 6 7 8 1 1 ...
## $ icd_code : chr "5723" "78959" "5715" "07070" ...
## $ icd_version: int 9 9 9 9 9 9 9 9 9 9 ...
## $ chartdate : IDate, format: NA ...
```

```
## $ age : num 52.3 52.3 52.3 52.3 52.3 ...
## $ admittime : POSIXct, format: "2180-05-06 22:23:00" ...
## $ enc_seq : int 1 1 1 1 1 1 1 1 1 2 ...
## $ cid : chr "10000032__22595853" "10000032__22595853"
##    "10000032__22595853" "10000032__22595853" ...
## - attr(*, ".internal.selfref")=<pointer: 0x105f63fc0>
## - attr(*, "sorted")= chr [1:3] "subject_id" "enc_seq" "hadm_id"
```

There are 7,365,770 total rows of data for 364,627 unique subjects and a total of 687,203 unique hospital encounters.

4 Investigating ICD Codes

The *medicalcoder* package provides utilities for investigating ICD codes. For the MIMIC-IV data, let's just check if the reported ICD codes are in the *medicalcoder* ICD data.

4.1 Utilities

There are several functions in the *medicalcoder* package for working with ICD codes:

- `get_icd_codes()`: returns a lookup table of ICD codes as a `data.frame`.
- `lookup_icd_codes()`: returns details on specific ICD codes.
- `is_icd()`: returns `TRUE` or `FALSE` for a vector of codes while considering ICD version, type, and billable status.
- `icd_compact_to_full()`: insert a decimal point into a string to be consistent with ICD-9 diagnostic, ICD-9 procedure, or ICD-10 diagnostic codes. (ICD-10 procedure codes do not have decimal places.) NOTE: this will not validate the return as a valid ICD code, just format the input string appropriately.

We encourage readers to review the vignette in *medicalcoder* for details on these utilities.

```
vignette(topic = "icd", package = "medicalcoder")
```

4.2 ICD-Codes in MIMIC-IV Data

Let's quickly verify that all the reported ICD codes in the MIMIC-IV data are known and ICD-9-CM, ICD-9-PCS, ICD-10-CM, or ICD-10-PCS codes.

```r
unique_icdcodes <-
  mimicivDT[
    !is.na(icd_code),
    unique(.SD),
    .SDcols = c("icd_code", "icd_version", "dx")
  ]
```

The call `medicalcoder::is_icd()` will return `TRUE` or `FALSE` for a vector of strings if the input is a known ICD code based on version (`icdv = 9` or `icdv = 10`), diagnostic (`dx = 1`) or procedure (`dx = 0`) codes, and source.

```r
# add the column `iscms`, need to call once for each pair of icdv/dx,
# This could be done with four explict calls or one with a by statement
unique_icdcodes[
  ,
  iscms := medicalcoder::is_icd(
             x    = icd_code,
             icdv = icd_version,
             dx   = dx,
             src  = "cms"
           ),
  by = .(icd_version, dx)
]
```

There are some codes that appear to not be valid ICD codes.

```r
unique_icdcodes[, .N, keyby = .(iscms)]
## Key: <iscms>
##     iscms     N
##    <lgcl> <int>
## 1:  FALSE    34
## 2:   TRUE 43460
```

Let's investigate these codes. All of them are header codes, never assignable.

```r
notcms <-
  merge(
    x = subset(medicalcoder::get_icd_codes(), src == "cms"),
    y = unique_icdcodes[!(iscms)],
    by.x = c('icdv', 'dx', 'code'),
    by.y = c('icd_version', 'dx', 'icd_code')
  )
head(notcms)
##   icdv dx code full_code src known_start known_end assignable_start
## 1   10  1  B96       B96 cms        2014      2026               NA
```

```
## 2   10   1 C722     C72.2 cms        2014       2026                NA
## 3   10   1  D40       D40 cms        2014       2026                NA
## 4   10   1  E11       E11 cms        2014       2026                NA
## 5   10   1  E66       E66 cms        2014       2026                NA
## 6   10   1 E760     E76.0 cms        2014       2026                NA
##   assignable_end iscms
## 1             NA FALSE
## 2             NA FALSE
## 3             NA FALSE
## 4             NA FALSE
## 5             NA FALSE
## 6             NA FALSE
all(is.na(notcms[["assignable_start"]]))
## [1] TRUE
```

If `headerok = TRUE` in the call `medicalcoder::is_icd()`, then these header codes will be considered valid.

```
unique_icdcodes[
  !is.na(icd_code),
  iscmshdrok := medicalcoder::is_icd(
                x = icd_code,
                icdv = icd_version,
                dx = dx,
                src = "cms",
                headerok = TRUE
              ),
  by = .(icd_version, dx)
  ]
unique_icdcodes[, .N, keyby = .(iscms, iscmshdrok)]
## Key: <iscms, iscmshdrok>
##     iscms iscmshdrok     N
##    <lgcl>     <lgcl> <int>
## 1:  FALSE       TRUE    34
## 2:   TRUE       TRUE 43460
```

Lastly, to get more detail on any of the codes you can use `medicalcoder::lookup_icd_codes()` or `medicalcoder::get_icd_codes()`.

```
medicalcoder::lookup_icd_codes(c("Z31", "S73109"))
##   input_code   match_type icdv dx full_code   code             src known_start
## 1     S73109 compact_code   10  1   S73.109 S73109             cms        2014
## 2        Z31    full_code   10  1       Z31    Z31             cms        2014
## 3        Z31    full_code   10  1       Z31    Z31          ihacpa        2020
## 4        Z31    full_code   10  1       Z31    Z31 socialstyrelsen        1997
## 5        Z31    full_code   10  1       Z31    Z31             who        2008
```

```
##   known_end assignable_start assignable_end
## 1      2026               NA             NA
## 2      2026               NA             NA
## 3      2026               NA             NA
## 4      2026               NA             NA
## 5      2021               NA             NA

subset(
  x = medicalcoder::get_icd_codes(with.description = TRUE),
  subset = code == "Z31"
)
##        icdv dx full_code code             src known_start known_end
## 343173   10  1       Z31  Z31             cms        2014      2026
## 343174   10  1       Z31  Z31 socialstyrelsen        1997      2026
## 343175   10  1       Z31  Z31          ihacpa        2020      2026
## 343176   10  1       Z31  Z31             who        2008      2021
##        assignable_start assignable_end                                desc
## 343173               NA             NA Encounter for procreative management
## 343174               NA             NA        Fertilitetsfrämjande åtgärder
## 343175               NA             NA               Procreative management
## 343176               NA             NA               Procreative management
##        desc_start desc_end
## 343173       2014     2026
## 343174       1997     2026
## 343175       2020     2026
## 343176       2008     2021

subset(
  x = medicalcoder::get_icd_codes(with.description = TRUE),
  subset = startsWith(code, "S73109")
)
##        icdv dx full_code    code src known_start known_end assignable_start
## 247110   10  1   S73.109  S73109 cms        2014      2026               NA
## 247111   10  1  S73.109A S73109A cms        2014      2026             2014
## 247112   10  1  S73.109D S73109D cms        2014      2026             2014
## 247113   10  1  S73.109S S73109S cms        2014      2026             2014
##        assignable_end
## 247110             NA
## 247111           2026
## 247112           2026
## 247113           2026
##                                                          desc desc_start
## 247110                       Unspecified sprain of unspecified hip       2014
## 247111    Unspecified sprain of unspecified hip, initial encounter       2014
## 247112 Unspecified sprain of unspecified hip, subsequent encounter       2014
## 247113             Unspecified sprain of unspecified hip, sequela       2014
##        desc_end
## 247110     2026
```

```
## 247111      2026
## 247112      2026
## 247113      2026
```

5 Flagging Encounter Level Comorbidities

In the following we will outline how to apply the comorbidities methods to the `mimicivDT` dataset, focusing on encounter level flagging of comorbidities. We start by showing how to apply the Charlson (Quan et al. 2005), Elixhauser (Quan et al. 2005), and PCCC v2 (Feudtner et al. 2014) methods to the `mimicivDT` dataset using the `medicalcoder::comorbidities()` call. Following that we will show how to do the same work using SQL code provided by MIMIC-code (Johnson et al. 2018), the R package *comorbidity* (Gasparini 2018), and the R package *pccc* (DeWitt et al. 2026). Lastly, we compare and contrast the differences in the flagged comorbidities between the methods.

5.1 `medicalcoder::comorbidities()`

A single function, `medicalcoder::comorbidities()`, is the interface for applying any of 21 comorbidity algorithms implemented by *medicalcoder*.

Usage: (Verbatim from *medicalcoder* package documentation)

```
comorbidities(
  data,
  icd.codes,
  method,
  id.vars = NULL,
  icdv.var = NULL,
  icdv = NULL,
  dx.var = NULL,
  dx = NULL,
  poa.var = NULL,
  poa = NULL,
  age.var = NULL,
  primarydx.var = NULL,
  primarydx = NULL,
  flag.method = c("current", "cumulative"),
  full.codes = TRUE,
  compact.codes = TRUE,
  subconditions = FALSE,
```

```
  mapping = c("precomputed", "regex")
)
```

Arguments: (Verbatim from *medicalcoder* package documentation)

`data` A `data.frame` in a "long" format. The input `data.frame` is expected to have one column of ICD codes (one code per row) with additional (optional) columns for patient/encounter ids, ICD version, diagnostic/procedure status, present-on-admission flags, primary diagnostic flags, or age.

`icd.codes` Character scalar naming the column in `data` that contains ICD codes (character strings). Codes may be provided in full form (with decimal points, e.g., C84.2), compact form (dots omitted, e.g., C842), or any mix of the two. Matching against lookup tables is governed by `icdv.var/icdv`, `dx.var/dx`, and the `full.codes / compact.codes` flags.

`method` Character string indicating the comorbidity algorithm to apply to `data`.

`id.vars` Optional character vector of column names. When missing, the entire input `data` is treated as a single encounter from a single patient. If you want to set `flag.method = "cumulative"` then `length(id.vars) >= 2` is expected. The last element should be the encounter order. It must not contain missing values, must not be a factor, and must be numeric, character, `Date`, or `POSIXt`. Character encounter order columns are allowed with a warning because they are sorted lexicographically.

`icdv.var` Character scalar naming the column in `data` that indicates the ICD version. If present it must be numeric. Rows with values other than `9L` or `10L` will not be used to map ICD codes to comorbidities. `icdv.var` takes precedence over `icdv` if both are provided.

`icdv` An integer value of `9L` or `10L` indicating that all `data[[icd.codes]]` are ICD version 9 or 10, respectively. Ignored (with a warning) if `icdv.var` is provided.

`dx.var` Character scalar naming the column in `data` that indicates diagnostic (`1L`) vs procedural (`0L`) codes. If present it must be numeric. Rows with values other than `0L` or `1L` will not be used to map ICD codes to comorbidities. `dx.var` takes precedence over `dx` if both are provided.

`dx` An integer indicating that all `data[[icd.codes]]` are diagnostic (`1L`) or procedure (`0L`) codes. Ignored (with a warning) if `dx.var` is provided.

`poa.var` Character scalar naming the column with present-on-admission flags: integer `1L` (present), `0L` (not present), or `NA`. For AHRQ SAS-compatible present-on-admission indicators, convert `"Y"` and `"W"` to `1L`, convert `"N"` and `"U"` to `0L`, and convert blank or missing values to `NA_integer_` before calling `comorbidities()`. PCCC and Charlson will only flag conditions when the code is present-on-admission. Elixhauser has a mix of conditions; some require present-on-admission while others do not. `poa.var` takes precedence over `poa` if both are provided.

poa Integer scalar `0L` or `1L`. Use when all `icd.codes` share the same present-on-admission status. Ignored with a warning if `poa` and `poa.var` are both provided.

age.var Character scalar naming the column in `data` that contains patient age in years. Only applicable to Charlson comorbidities.

primarydx.var Character scalar naming the column in `data` that indicates whether `data[[icd.codes]]` are primary diagnostic codes (`1L`) or not (`0L`). Primary diagnosis is used only for Elixhauser and Charlson comorbidities and is ignored when the method is a PCCC variant. `primarydx.var` takes precedence over `primarydx` if both are provided.

primarydx An integer value of `0L` or `1L`. If `0L`, treat all codes as non-primary diagnoses; if `1L`, treat all codes as primary diagnoses. Ignored, with a warning, if `primarydx.var` is provided.

flag.method When `flag.method = "current"` (default) only codes associated with the current `id.vars` are considered when flagging comorbidities. The `flag.method = "cumulative"` option also considers prior encounters when flagging comorbidities. See **Details**.

full.codes, compact.codes Logical; when `TRUE` compare `data[[icd.codes]]` against full and/or compact ICD codes in the method's lookup tables. Full ICD codes include a decimal point (when applicable) and compact codes omit the decimal point. For example: `B95.0` is the full ICD-10-CM diagnostic code for "Streptococcus, group A, as the cause of disease classified elsewhere," whereas `B950` is the associated compact code.

subconditions Logical scalar; when `TRUE`, report both conditions and subconditions (PCCC only).

mapping Character string specifying how `data[[icd.codes]]` should be mapped to comorbidity conditions. `mapping = "precomputed"` uses the precomputed ICD code-condition links included with medicalcoder and is the default. `mapping = "regex"` applies the method's regular expressions directly to the input ICD codes. See Details.

5.1.1 Charlson Comorbidities

There are several variants of Charlson like comorbidity methods implemented in *medicalcoder*. One way to see the current list of implemented methods is to look at the names of the lookup table mapping ICD codes to conditions:

```
grep(
  pattern = "^charlson",
  x = names(medicalcoder::get_charlson_codes()),
  value = TRUE
)
## [1] "charlson_beyrer2021"       "charlson_cdmf2019"
```

```
## [3] "charlson_deyo1992"          "charlson_ludvigsson2021"
## [5] "charlson_mimicivcode"       "charlson_quan2005"
## [7] "charlson_sundararajan2004" "charlson_quan2011"
```

The `charlson_quan2011` method will be applied to the `mimicivDT` dataset and the results will be compared to the results from the `comorbidity::comorbidity()` method. These are the same ICD code mappings as `charlson_quan2005`. The difference is the weights used for the Charlson Comorbidity Index calculation.

```
medicalcoder_charlson_quan2011 <-
  medicalcoder::comorbidities(
    data        = mimicivDT,  # object inheriting data.frame
    icd.codes   = "icd_code", # character string name of icd codes in data
    id.vars     = c("subject_id", "hadm_id"),
    icdv.var    = "icd_version",
    dx.var      = "dx",
    poa         = 1L, # consider all codes present on admission
    primarydx   = 0L, # consider all diagnosis codes secondary diagnoses.
    method      = "charlson_quan2011",
    flag.method = "current" # default
  )
data.table::setkey(medicalcoder_charlson_quan2011, subject_id, hadm_id)
```

The SQL code for applying Charlson comorbidities to the MIMIC-IV data provided by MIT-LCP has a slightly different set of ICD-10 codes mapping to conditions. Quan et al. (2005, 2011) is based on ICD-10 codes, not ICD-10-CM. The MIMIC-IV SQL code includes the C4A category which is an ICD-10-CM specific category (see this github issue). To be consistent with the expected set of ICD codes identified in the Quan papers and the MIMIC-IV SQL code, *medicalcoder* provides distinct methods.

```
medicalcoder_charlson_mimiciv <-
  medicalcoder::comorbidities(
    data        = mimicivDT,
    icd.codes   = "icd_code",
    id.vars     = c("subject_id", "hadm_id"),
    icdv.var    = "icd_version",
    dx.var      = "dx",
    age.var     = "age",
    poa         = 1L,
    primarydx   = 0L,
    method      = "charlson_mimicivcode",
  )
data.table::setkey(medicalcoder_charlson_mimiciv, subject_id, hadm_id)
```

5.1.2 Elixhauser Comorbidities

As with the Charlson comorbidities there are multiple variants of Elixhauser like comorbidity methods implemented in *medicalcoder*.

```
grep(
  pattern = "^elixhauser",
  x = names(medicalcoder::get_elixhauser_codes()),
  value = TRUE
)
## [1] "elixhauser_ahrq_web"       "elixhauser_elixhauser1988"
## [3] "elixhauser_quan2005"       "elixhauser_ahrq2022"
## [5] "elixhauser_ahrq2023"       "elixhauser_ahrq2024"
## [7] "elixhauser_ahrq2025"       "elixhauser_ahrq2026"
## [9] "elixhauser_ahrq_icd10"
```

To apply the `elixhauser_quan2005` version, which is comparable to the R package *comorbidity*, do the following:

```
medicalcoder_elixhauser_quan2005 <-
  medicalcoder::comorbidities(
    data        = mimicivDT,
    icd.codes   = "icd_code",
    id.vars     = c("subject_id", "hadm_id"),
    icdv.var    = "icd_version",
    dx.var      = "dx",
    poa         = 1L,
    primarydx   = 0L,
    method      = "elixhauser_quan2005"
  )
data.table::setkey(medicalcoder_elixhauser_quan2005, subject_id, hadm_id)
```

5.1.3 PCCC v2.0

PCCC v2.0 has been implemented to be consistent with the R package *pccc*. The v2.1 variants extends the ICD to conditions mapping while using the same underlying algorithm. v3.0 and v3.1 use a different set of ICD codes and algorithm for flagging conditions. For this work we focus on v2.0 so we can compare it to the results from the R package *pccc*.

```
medicalcoder_pccc_v2.0 <-
  medicalcoder::comorbidities(
    data        = mimicivDT,
    icd.codes   = "icd_code",
    id.vars     = c("subject_id", "hadm_id"),
```

```
    icdv.var    = "icd_version",
    dx.var      = "dx",
    poa         = 1L,
    method      = "pccc_v2.0"
  )
data.table::setkey(medicalcoder_pccc_v2.0, subject_id, hadm_id)
```

5.2 MIMIC-IV Code

We use the SQL code for applying the Charlson comorbidity algorithm to the MIMIC-IV data provided by the MIT Laboratory for Computational Physiology (MIT_LCP) on GitHub. We stored the path to a local copy of the repository in the system environmental variable `MIMICIVCODE`. The specific version of the SQL code being used is from commit 278df75ec30991ff3a6f5ceb6d2221635a085e9f

```
mimiciv_charlson_sql <-
  file.path(Sys.getenv("MIMICIVCODE"), "concepts", "comorbidity", "charlson.sql")

# checksum
stopifnot(
  substr(
    system(
      sprintf("sha256sum %s", mimiciv_charlson_sql),
      intern = TRUE
    ),
  start = 1L,
  stop = 64L
  ) == "5b797b673ace4dcbc6f686848d24371382f49ac61fcc39101f09c3b42969d801"
)
```

The SQL code is expected to run on Google Big Query. We make a few small modifications to the code so we can evaluate the SQL locally via RSQLite.

```
# scan in the sql code
mimiciv_charlson_sql <-
  scan(
    file = mimiciv_charlson_sql,
    what = "character",
    sep = "\n",
    quiet = TRUE
  )

# modify the query to work in SQLite
# replace Google Big Query table names with table names to be using in
```

```r
# the local RSQLite in memory database.  Three table names need to be
# changed and one function call: GBQ GREATEST needs to be replaced by MAX
prs <-
  c(
    "physionet-data.mimiciv_hosp.admissions" = "admissions",
    "physionet-data.mimiciv_hosp.diagnoses_icd" = "diagnoses",
    "physionet-data.mimiciv_derived.age" = "ages",
    "GREATEST" = "MAX"
  )
for(i in seq_len(length(prs))) {
  mimiciv_charlson_sql <-
    gsub(
      pattern     = names(prs)[i],
      replacement = prs[i],
      x           = mimiciv_charlson_sql,
      fixed       = TRUE
    )
}

mimiciv_charlson_sql <- paste(mimiciv_charlson_sql, collapse = "\n")
```

We set up an SQL database in memory and evaluate the code.

```r
con <- odbc::dbConnect(drv = RSQLite::SQLite(), dbname = ":memory:")

# add data to the data base
with(mimicivdata, {
  odbc::dbWriteTable(conn = con, name = "diagnoses",  value = diagnoses_icd)
  odbc::dbWriteTable(conn = con, name = "admissions", value = admissions)
  odbc::dbWriteTable(conn = con, name = "patients",   value = patients)
  odbc::dbWriteTable(conn = con, name = "ages",       value = ages)
})

# Run the query and record the time required to do so
mimiciv_charlson <- odbc::dbGetQuery(con, mimiciv_charlson_sql)
# close DB connection
odbc::dbDisconnect(conn = con)
data.table::setDT(mimiciv_charlson)
data.table::setkey(mimiciv_charlson, subject_id, hadm_id)
```

5.3 `comorbidity::comorbidity()`

The R package *comorbidity* (Gasparini 2018) is a widely used and referenced tool for applying the Charlson and Elixhauser comorbidities. One consideration for using `comorbidity::comorbidity()` is that ICD-9 or ICD-10 codes are used independently. There

are 30921 subjects with both ICD-9 and ICD-10 codes in their records, and 11 encounters with both ICD versions.

To flag the conditions appropriately we must split the data by ICD version, apply `comorbidity::comorbidity()` to each split, and then aggregate the results. Also, `comorbidity::comorbidity()` only takes one column for the id and thus we need to use `cid` for the id instead of the `c("subject_id", "hadm_id")` used in the calls to `medicalcoder::comorbidities()`. In the following work we will copy the data built for the encounter level flagging and extend for cumulative flagging as well.

The following is done using *comorbidity* version 1.1.0.

5.3.1 Charlson

```
comorbidity_charlson_icd9 <-
  comorbidity::comorbidity(
    x       = subset(mimicivDT, icd_version == 9 & dx == 1L),
    id      = "cid",
    code    = "icd_code",
    map     = "charlson_icd9_quan",
    assign0 = TRUE # set less severe comorbidities flags to 0 when more
                   # severe comorbidities is also flagged
  )

comorbidity_charlson_icd10 <-
  comorbidity::comorbidity(
    x       = subset(mimicivDT, icd_version == 10 & dx == 1L),
    id      = "cid",
    code    = "icd_code",
    map     = "charlson_icd10_quan",
    assign0 = TRUE
  )

comorbidity_charlson <-
  rbind(
    comorbidity_charlson_icd9,
    comorbidity_charlson_icd10
  )

# aggregate between the icd versions
comorbidity_charlson <-
  aggregate(. ~ cid, data = comorbidity_charlson, FUN = max)

# apply the scoring, to use comorbidity::score the object needs to have certain
# attributes which were lost when aggregating over the icd versions.  Replace
```

```r
# the attributes
attributes(comorbidity_charlson)[c("class", "variable.labels", "map")] <-
  attributes(comorbidity_charlson_icd9)[c("class", "variable.labels", "map")]

comorbidity_charlson[["score"]] <-
  comorbidity::score(
    x = comorbidity_charlson,
    weights = "quan",
    assign0 = TRUE
  )

# expand the id columns
data.table::setDT(comorbidity_charlson)
comorbidity_charlson[
  ,
  c("subject_id", "hadm_id") :=
    lapply(data.table::tstrsplit(cid, "__"), as.integer)
  ]
data.table::set(comorbidity_charlson, j = "cid", value = NULL)
data.table::setkey(comorbidity_charlson, subject_id, hadm_id)
```

5.3.2 Elixhauser

Compared to the work done for flagging Charlson comorbidities, Elixhauser flagging requires changing the `map` argument in the calls to `comorbidity::comorbidity()` and there is no scoring to be done.

```r
comorbidity_elixhauser_icd9 <-
  comorbidity::comorbidity(
    x       = subset(mimicivDT, icd_version == 9 & dx == 1L),
    id      = "cid",
    code    = "icd_code",
    map     = "elixhauser_icd9_quan",
    assign0 = TRUE
  )

comorbidity_elixhauser_icd10 <-
  comorbidity::comorbidity(
    x       = subset(mimicivDT, icd_version == 10 & dx == 1L),
    id      = "cid",
    code    = "icd_code",
    map     = "elixhauser_icd10_quan",
    assign0 = TRUE
  )

comorbidity_elixhauser <-
```

```
  rbind(
    comorbidity_elixhauser_icd9,
    comorbidity_elixhauser_icd10
  )

# aggregate between the icd versions
comorbidity_elixhauser <-
  aggregate(. ~ cid, data = comorbidity_elixhauser, FUN = max)

# expand the id columns
data.table::setDT(comorbidity_elixhauser)
comorbidity_elixhauser[
  ,
  c("subject_id", "hadm_id") :=
    lapply(data.table::tstrsplit(cid, "__"), as.integer)
  ]
data.table::set(comorbidity_elixhauser, j = "cid", value = NULL)
data.table::setkey(comorbidity_elixhauser, subject_id, hadm_id)
```

5.4 `pccc::ccc()`

The R package *pccc* (DeWitt et al. 2026) implements PCCC v2 (Feudtner et al. 2014). As with `comorbidity::comorbidity()` and many other implementations of comorbidities, only one version of ICD is assessed at a time.

The expected input structure of the data for `pccc::ccc()` is a wide format, one row per encounter, one column for each ICD code. The following uses *pccc* version 1.0.7.

```
mimicivDT_for_pccc <-
  split(mimicivDT, by = "icd_version") |>
  lapply(
    data.table::dcast,
    formula = cid ~ ifelse(dx == 1, "DX", "PR") + seq_num,
    value.var = "icd_code"
  )

pccc_pcccv2.0 <-
  rbind(
    pccc::ccc(
      data = mimicivDT_for_pccc[["9"]],
      id = cid,
      dx_cols = grep("^DX", names(mimicivDT_for_pccc[["9"]]), value = TRUE),
      pc_cols = grep("^PR", names(mimicivDT_for_pccc[["9"]]), value = TRUE),
      icdv = 9
    )
    ,
```

```
    pccc::ccc(
      data = mimicivDT_for_pccc[["10"]],
      id = cid,
      dx_cols = grep("^DX", names(mimicivDT_for_pccc[["10"]]), value = TRUE),
      pc_cols = grep("^PR", names(mimicivDT_for_pccc[["10"]]), value = TRUE),
      icdv = 10
    )
  )

# aggregate over ICD version
pccc_pcccv2.0 <- aggregate(. ~ cid, data = pccc_pcccv2.0, FUN = max)

# expand the id columns
data.table::setDT(pccc_pcccv2.0)
pccc_pcccv2.0[
  ,
  c("subject_id", "hadm_id") :=
    lapply(data.table::tstrsplit(cid, "__"), as.integer)
  ]
data.table::set(pccc_pcccv2.0, j = "cid", value = NULL)
data.table::setkey(pccc_pcccv2.0, subject_id, hadm_id)
```

6 Comparing Encounter-level Flags Between Utilities

6.1 Charlson Comorbidities

6.1.1 `medicalcoder::comorbidities()` vs `comorbidity::comorbidity()`

The first thing to note is that the number of rows returned by the two implementations is different.

```
nrow(medicalcoder_charlson_quan2011)
## [1] 687203
nrow(comorbidity_charlson)
## [1] 545497
```

The reason for this is that there are encounters with no diagnostic ICD codes. `medicalcoder::comorbidities()` returns a row for these encounters and `comorbidity::comorbidity()` does not. For these encounters, `medicalcoder::comorbidities()` returns a row with zero comorbidities.

```r
# subject/hadm_id not in comorbidity::comorbidity() results all have no
# diagnostic codes.
mimicivDT[!comorbidity_charlson][, any(dx == 1 & !is.na(icd_code))]
## [1] TRUE
medicalcoder_charlson_quan2011[!comorbidity_charlson][, all(cmrb_flag == 0L)]
## [1] TRUE
```

We build the `mdcr_v_cmrb` object to make comparing the results between the `medicalcoder::comorbidities()` call and the `comorbidity::comorbidity()` call easier. We will only include the `subject_id`/`hadm_id` reported on by both.

```r
mdcr_v_cmrb <-
  merge(
    x = medicalcoder_charlson_quan2011,
    y = comorbidity_charlson,
    all = FALSE,
    by = c("subject_id", "hadm_id"),
    suffixes = c("_mdcr", "_cmrb")
  )
```

The relevant columns to compare are as follows:

```r
mvc_columns <-
  data.table::fread(text = "
    medicalcoder   | comorbidity
    aidshiv        | aids
    cebvd          | cevd
    chf_mdcr       | chf_cmrb
    hp_mdcr        | hp_cmrb
    copd           | cpd
    dem            | dementia
    dm             | diab
    dmc            | diabwc
    mal            | canc
    mi_mdcr        | mi_cmrb
    mld_mdcr       | mld_cmrb
    msld_mdcr      | msld_cmrb
    mst            | metacanc
    pud_mdcr       | pud_cmrb
    pvd_mdcr       | pvd_cmrb
    rhd            | rheumd
    rnd            | rend
    cci            | score
  ")
```

If the columns are identical, we remove them from the `mdcr_v_cmrb` object. This test includes the class of the column. `medicalcoder::comorbidities()` returns integer values as does `comorbidity::comorbidity()` save the `score` columns, which are numeric.

```
data.table::set(
  x = mdcr_v_cmrb,
  j = "score",
  value = as.integer(mdcr_v_cmrb[["score"]])
)

for (i in seq_len(nrow(mvc_columns))) {
  x <- mvc_columns[["medicalcoder"]][i]
  y <- mvc_columns[["comorbidity"]][i]
  z <- identical(mdcr_v_cmrb[[x]], mdcr_v_cmrb[[y]])
  if (z) {
    message(sprintf("`%s` and `%s` are identical.", x, y))
    data.table::set(mdcr_v_cmrb, j = x, value = NULL)
    data.table::set(mdcr_v_cmrb, j = y, value = NULL)
  } else {
    stop(sprintf("`%s` and `%s` are not identical.", x, y))
  }
}
## `aidshiv` and `aids` are identical.
## `cebvd` and `cevd` are identical.
## `chf_mdcr` and `chf_cmrb` are identical.
## `hp_mdcr` and `hp_cmrb` are identical.
## `copd` and `cpd` are identical.
## `dem` and `dementia` are identical.
## `dm` and `diab` are identical.
## `dmc` and `diabwc` are identical.
## `mal` and `canc` are identical.
## `mi_mdcr` and `mi_cmrb` are identical.
## `mld_mdcr` and `mld_cmrb` are identical.
## `msld_mdcr` and `msld_cmrb` are identical.
## `mst` and `metacanc` are identical.
## `pud_mdcr` and `pud_cmrb` are identical.
## `pvd_mdcr` and `pvd_cmrb` are identical.
## `rhd` and `rheumd` are identical.
## `rnd` and `rend` are identical.
## `cci` and `score` are identical.
```

The only columns remaining in the `mdcr_v_cmrb` object are id columns and three columns unique to the return from `medicalcoder::comorbidities()`: * `num_cmrb`: the number of flagged comorbidities, * `cmrb_flag`: a comorbidity flag (at least one comorbidity), and * `age_score`: an age score (all `NA`) in this case.

```r
str(mdcr_v_cmrb)
## Classes 'medicalcoder_comorbidities', 'data.table' and 'data.frame': 545497 obs. of  5 variables:
## $ subject_id: int 10000032 10000032 10000032 10000032 10000068 10000084
##    10000084 10000108 10000117 10000117 ...
## $ hadm_id : int 22595853 22841357 25742920 29079034 25022803 23052089 29888819
##    27250926 22927623 27988844 ...
## $ num_cmrb : int 2 2 2 2 0 1 1 0 0 0 ...
## $ cmrb_flag : int 1 1 1 1 0 1 1 0 0 0 ...
## $ age_score : int NA NA NA NA NA NA NA NA NA NA ...
## - attr(*, "sorted")= chr [1:2] "subject_id" "hadm_id"
## - attr(*, ".internal.selfref")=<pointer: 0x105f63fc0>
```

6.1.2 `medicalcoder::comorbidities()` vs MIMIC-IV Code

There are some noteable structural differnces between the results from `medicalcoder::comorbidities()` and the MIMIC-IV Code. First, note that the nubmer of rows differ. This time for different reasons than when we saw different results between `medicalcoder::comorbidities()` and `comorbidity::comorbidity()`.

```r
nrow(medicalcoder_charlson_mimiciv)
## [1] 687203
nrow(mimiciv_charlson)
## [1] 546028
```

These records with no comorbidities are not returned by the MIMIC-IV Code. More precisely, all subject/hadm_id pairs missing from the MIMIC-IV Code have a missing `hadm_id`.

```r
mimicivDT[!mimiciv_charlson, on = c("hadm_id", "hadm_id")][, all(is.na(hadm_id))]
## [1] TRUE
```

Build the `mdcr_v_mmcc` object to make comparing the results between the `medicalcoder::comorbidities()` call and the MIMIC-IV SQL easier.

```r
mdcr_v_mmcc <-
  merge(
    x = medicalcoder_charlson_mimiciv,
    y = mimiciv_charlson,
    all = FALSE,
    by = c("subject_id", "hadm_id"),
    suffixes = c("_mdcr", "_mimiciv")
  )
```

Another difference in the output from the MIMIC-IV SQL and `medicalcoder::comorbidities()` is that when an encounter has ICD codes for the less severe and more severe version of a condition the MIMIC-IV code reports the flag for both whereas `medicalcoder::comorbidities()` only returns the flag for the more severe case. We set the less severe case for the MIMIC-IV code results to zero when the more severe case is present before comparing all the results.

```
# Set the MIMIC-IV code DM without cc to 0 when cc exist
mdcr_v_mmcc[
  diabetes_without_cc == 1L & diabetes_with_cc == 1L,
  diabetes_without_cc := 0L
]

# Set mild liver disease to 0 when sever liver disease exits for MIMIC-IV code
# results
mdcr_v_mmcc[
  mild_liver_disease == 1L & severe_liver_disease == 1L,
  mild_liver_disease := 0L
]

# set malignant_cancer to zero when metastatic_solid_tumor exits for MIMIC-IV
# code results
mdcr_v_mmcc[
  malignant_cancer == 1L & metastatic_solid_tumor == 1L,
  malignant_cancer := 0L
]
```

Now that the flags have been adjusted, the relevant columns to compare between `medicalcoder::comorbidities()` and the MIMIC-IV Code are:

```
mvm_columns <-
  data.table::fread(text = "
    medicalcoder    | mimicivcode
    aidshiv         | aids
    cebvd           | cerebrovascular_disease
    chf             | congestive_heart_failure
    copd            | chronic_pulmonary_disease
    dem             | dementia
    dm              | diabetes_without_cc
    dmc             | diabetes_with_cc
    hp              | paraplegia
    mal             | malignant_cancer
    mi              | myocardial_infarct
    mld             | mild_liver_disease
    msld            | severe_liver_disease
    mst             | metastatic_solid_tumor
    pud             | peptic_ulcer_disease
    pvd             | peripheral_vascular_disease
```

```
    rhd           | rheumatic_disease
    rnd           | renal_disease
    age_score_mdcr | age_score_mimiciv
    cci           | charlson_comorbidity_index
  ")
```

If the columns are identical, they are removed from the `mdcr_v_mmcc` object.

```
for (i in seq_len(nrow(mvm_columns))) {
  x <- mvm_columns[["medicalcoder"]][i]
  y <- mvm_columns[["mimicivcode"]][i]
  z <- identical(mdcr_v_mmcc[[x]], mdcr_v_mmcc[[y]])
  if (z) {
    message(sprintf("`%s` and `%s` are identical.", x, y))
    data.table::set(mdcr_v_mmcc, j = x, value = NULL)
    data.table::set(mdcr_v_mmcc, j = y, value = NULL)
  } else {
    stop(sprintf("`%s` and `%s` are not identical.", x, y))
  }
}
## `aidshiv` and `aids` are identical.
## `cebvd` and `cerebrovascular_disease` are identical.
## `chf` and `congestive_heart_failure` are identical.
## `copd` and `chronic_pulmonary_disease` are identical.
## `dem` and `dementia` are identical.
## `dm` and `diabetes_without_cc` are identical.
## `dmc` and `diabetes_with_cc` are identical.
## `hp` and `paraplegia` are identical.
## `mal` and `malignant_cancer` are identical.
## `mi` and `myocardial_infarct` are identical.
## `mld` and `mild_liver_disease` are identical.
## `msld` and `severe_liver_disease` are identical.
## `mst` and `metastatic_solid_tumor` are identical.
## `pud` and `peptic_ulcer_disease` are identical.
## `pvd` and `peripheral_vascular_disease` are identical.
## `rhd` and `rheumatic_disease` are identical.
## `rnd` and `renal_disease` are identical.
## `age_score_mdcr` and `age_score_mimiciv` are identical.
## `cci` and `charlson_comorbidity_index` are identical.
```

The only remaining columns are id columns and the `num_cmrb` column, which is unique to the return from `medicalcoder::comorbidities()`.

```
str(mdcr_v_mmcc)
## Classes 'medicalcoder_comorbidities', 'data.table' and 'data.frame': 546028 obs. of  4 variables:
## $ subject_id: int 10000032 10000032 10000032 10000032 10000068 10000084
```

```
##    10000084 10000108 10000117 10000117 ...
## $ hadm_id : int 22595853 22841357 25742920 29079034 25022803 23052089 29888819
##    27250926 22927623 27988844 ...
## $ num_cmrb : int 2 2 2 2 0 1 1 0 0 0 ...
## $ cmrb_flag : int 1 1 1 1 0 1 1 0 0 0 ...
## - attr(*, "sorted")= chr [1:2] "subject_id" "hadm_id"
## - attr(*, ".internal.selfref")=<pointer: 0x105f63fc0>
## - attr(*, "index")= int(0)
```

6.2 Elixhauser Comorbidities

6.2.1 `medicalcoder::comorbidities()` vs `comorbidity::comorbidity()`

As with the Charlson comparison, the numbers of rows between the two results differ. And for the same reason: no diagnostic ICD codes.

```
nrow(medicalcoder_elixhauser_quan2005)
## [1] 687203
nrow(comorbidity_elixhauser)
## [1] 545497
# subject/hadm_id not in comorbibity::comorbibity() results all have no
# diagnostic codes.
mimicivDT[!comorbidity_elixhauser][, any(dx == 1 & !is.na(icd_code))]
## [1] TRUE
medicalcoder_elixhauser_quan2005[!comorbidity_elixhauser][, all(cmrb_flag == 0L)]
## [1] TRUE
```

We build the `mdcr_v_cmrb_elix` object to make comparing the results between the `medicalcoder::comorbidities()` call and the `comorbidity::comorbidity()` call easier. We will only include the `subject_id`/`hadm_id` reported on by both.

```
mdcr_v_cmrb_elix <-
  merge(
    x = medicalcoder_elixhauser_quan2005,
    y = comorbidity_elixhauser,
    all = FALSE,
    by = c("subject_id", "hadm_id"),
    suffixes = c("_mdcr", "_cmrb")
  )
```

The relevant columns to compare are as follows:

```r
mvc_elix_columns <-
  data.table::fread(text = "
    medicalcoder        | comorbidity
    AIDS                | aids
    ALCOHOL             | alcohol
    ANEMDEF             | dane
    ARTH                | rheumd
    BLDLOSS             | blane
    CARDIAC_ARRHYTHMIAS | carit
    CHF                 | chf
    CHRNLUNG            | cpd
    COAG                | coag
    DEPRESS             | depre
    DM                  | diabunc
    DMCX                | diabc
    DRUG                | drug
    HTN_CX              | hypc
    HTN_UNCX            | hypunc
    HYPOTHY             | hypothy
    LIVER               | ld
    LYMPH               | lymph
    LYTES               | fed
    METS                | metacanc
    NEURO               | ond
    OBESE               | obes
    PARA                | para
    PERIVASC            | pvd
    PSYCH               | psycho
    PULMCIRC            | pcd
    RENLFAIL            | rf
    TUMOR               | solidtum
    ULCER               | pud
    VALVE               | valv
    WGHTLOSS            | wloss
  ")
```

If the columns are identical, we remove them from the `mdcr_v_cmrb_elix` object.

```r
for (i in seq_len(nrow(mvc_elix_columns))) {
  x <- mvc_elix_columns[["medicalcoder"]][i]
  y <- mvc_elix_columns[["comorbidity"]][i]
  z <- identical(mdcr_v_cmrb_elix[[x]], mdcr_v_cmrb_elix[[y]])
  if (z) {
    message(sprintf("`%s` and `%s` are identical.", x, y))
    data.table::set(mdcr_v_cmrb_elix, j = x, value = NULL)
    data.table::set(mdcr_v_cmrb_elix, j = y, value = NULL)
  } else {
```

```
    stop(sprintf("`%s` and `%s` are not identical.", x, y))
  }
}
## `AIDS` and `aids` are identical.
## `ALCOHOL` and `alcohol` are identical.
## `ANEMDEF` and `dane` are identical.
## `ARTH` and `rheumd` are identical.
## `BLDLOSS` and `blane` are identical.
## `CARDIAC_ARRHYTHMIAS` and `carit` are identical.
## `CHF` and `chf` are identical.
## `CHRNLUNG` and `cpd` are identical.
## `COAG` and `coag` are identical.
## `DEPRESS` and `depre` are identical.
## `DM` and `diabunc` are identical.
## `DMCX` and `diabc` are identical.
## `DRUG` and `drug` are identical.
## `HTN_CX` and `hypc` are identical.
## `HTN_UNCX` and `hypunc` are identical.
## `HYPOTHY` and `hypothy` are identical.
## `LIVER` and `ld` are identical.
## `LYMPH` and `lymph` are identical.
## `LYTES` and `fed` are identical.
## `METS` and `metacanc` are identical.
## `NEURO` and `ond` are identical.
## `OBESE` and `obes` are identical.
## `PARA` and `para` are identical.
## `PERIVASC` and `pvd` are identical.
## `PSYCH` and `psycho` are identical.
## `PULMCIRC` and `pcd` are identical.
## `RENLFAIL` and `rf` are identical.
## `TUMOR` and `solidtum` are identical.
## `ULCER` and `pud` are identical.
## `VALVE` and `valv` are identical.
## `WGHTLOSS` and `wloss` are identical.
```

The only columns remaining in the `mdcr_v_cmrb_elix` object are id columns and columns unique to the return from `medicalcoder::comorbidities()`: * `num_cmrb`: the number of flagged comorbidities, * `cmrb_flag`: a comorbidity flag (at least one comorbidity), * `HTN_C`: any hypertension, * `mortality_index` and `readmission_index`: based on the weights from AHRQ.

```
str(mdcr_v_cmrb_elix)
## Classes 'medicalcoder_comorbidities', 'data.table' and 'data.frame': 545497 obs. of  7 variables:
## $ subject_id : int 10000032 10000032 10000032 10000032 10000068 10000084
##    10000084 10000108 10000117 10000117 ...
## $ hadm_id : int 22595853 22841357 25742920 29079034 25022803 23052089 29888819
```

```
##    27250926 22927623 27988844 ...
## $ HTN_C : int 0 0 0 0 0 0 0 0 0 0 ...
## $ num_cmrb : int 3 4 3 4 1 1 1 0 1 1 ...
## $ cmrb_flag : int 1 1 1 1 1 1 1 0 1 1 ...
## $ mortality_index : int 2 29 18 27 -1 5 5 0 0 0 ...
## $ readmission_index: int 22 33 26 36 6 7 7 0 0 0 ...
## - attr(*, "sorted")= chr [1:2] "subject_id" "hadm_id"
## - attr(*, ".internal.selfref")=<pointer: 0x105f63fc0>
```

6.3 PCCC

6.3.1 `medicalcoder::comorbidities()` vs `pccc::ccc()`

As we have seen in other cases, the absence of ICD codes for a `hadm_id` results in the omission of the record from the return from `pccc::ccc()`.

```
nrow(medicalcoder_pccc_v2.0)
## [1] 687203
nrow(pccc_pcccv2.0)
## [1] 545576
```

For the common `subject_id`/`hadm_id` pairs we compare the flags.

```
mdcr_v_pccc <-
  merge(
    x = medicalcoder_pccc_v2.0,
    y = pccc_pcccv2.0,
    all = FALSE,
    by = c("subject_id", "hadm_id"),
    suffixes = c("_mdcr", "_pccc")
  )
```

```
mvp_columns <- data.table::fread(text = "
  medicalcoder | pccc
  congeni_genetic_mdcr | congeni_genetic_pccc
  cvd_mdcr | cvd_pccc
  gi_mdcr | gi_pccc
  hemato_immu_mdcr | hemato_immu_pccc
  malignancy_mdcr | malignancy_pccc
  metabolic_mdcr | metabolic_pccc
  neonatal_mdcr | neonatal_pccc
  neuromusc_mdcr | neuromusc_pccc
  renal_mdcr | renal_pccc
  respiratory_mdcr | respiratory_pccc
```

```
  cmrb_flag | ccc_flag"
)
```

```
for (i in seq_len(nrow(mvp_columns))) {
  x <- mvp_columns[["medicalcoder"]][i]
  y <- mvp_columns[["pccc"]][i]
  z <- identical(mdcr_v_pccc[[x]], mdcr_v_pccc[[y]])
  if (z) {
    message(sprintf("`%s` and `%s` are identical.", x, y))
    data.table::set(mdcr_v_pccc, j = x, value = NULL)
    data.table::set(mdcr_v_pccc, j = y, value = NULL)
  } else {
    stop(sprintf("`%s` and `%s` are not identical.", x, y))
  }
}
## `congeni_genetic_mdcr` and `congeni_genetic_pccc` are identical.
## `cvd_mdcr` and `cvd_pccc` are identical.
## `gi_mdcr` and `gi_pccc` are identical.
## `hemato_immu_mdcr` and `hemato_immu_pccc` are identical.
## `malignancy_mdcr` and `malignancy_pccc` are identical.
## `metabolic_mdcr` and `metabolic_pccc` are identical.
## `neonatal_mdcr` and `neonatal_pccc` are identical.
## `neuromusc_mdcr` and `neuromusc_pccc` are identical.
## `renal_mdcr` and `renal_pccc` are identical.
## `respiratory_mdcr` and `respiratory_pccc` are identical.
## `cmrb_flag` and `ccc_flag` are identical.
```

There are some differences in the results between the two approaches.

```
str(mdcr_v_pccc)
## Classes 'medicalcoder_comorbidities', 'data.table' and 'data.frame': 545576 obs. of  8 variables:
## $ subject_id : int 10000032 10000032 10000032 10000032 10000068 10000084
##    10000084 10000108 10000117 10000117 ...
## $ hadm_id : int 22595853 22841357 25742920 29079034 25022803 23052089 29888819
##    27250926 22927623 27988844 ...
## $ misc : int 0 0 1 1 0 0 0 0 0 0 ...
## $ any_tech_dep : int 0 0 1 1 0 0 0 0 0 0 ...
## $ any_transplant: int 0 0 0 0 0 0 0 0 0 0 ...
## $ num_cmrb : int 1 2 3 3 0 2 2 0 1 1 ...
## $ tech_dep : int 0 0 1 1 0 0 0 0 0 0 ...
## $ transplant : int 0 0 0 0 0 0 0 0 0 0 ...
## - attr(*, "sorted")= chr [1:2] "subject_id" "hadm_id"
## - attr(*, ".internal.selfref")=<pointer: 0x105f63fc0>
mdcr_v_pccc[
  ,
  .(discordant_n = sum(any_transplant != transplant | any_tech_dep != tech_dep), .N)
```

```
  ][
  ,
  .(discordant_n, discordant_p = discordant_n / N)
  ]
##    discordant_n discordant_p
##           <int>        <num>
## 1:         5476    0.0100371
```

The `misc` column is the "miscellaneous" category reported by `medicalcoder::comorbidities()` and is not reported by `pccc::ccc()`. The existence of the `misc` column and some differences in the returned results between `pccc::ccc()` version 1.0.7, and `medicalcoder::comorbidities()` is due to how medicalcoder is implemented.

The differences in any technology dependence and transplant are due to design difference between *medicalcoder* and *pccc*. *medicalcoder* uses database style joins to map precomputed ICD codes to conditions. This allows for a simple extension of mapping ICD codes to both the primary condition and subcondition, as the latter requires only an additional column in a lookup table. The *pccc* uses partial string matching to map ICD codes to conditions for each of the primary conditions and only the two subconditions of technology dependence and transplant. When building *medicalcoder*, the lookup table with condition and subconditions was extended to match the publication documentation.

```
mdcr_v_pccc[, .N, keyby = .(medicalcoder = any_tech_dep, pccc = tech_dep)]
## Key: <medicalcoder, pccc>
##    medicalcoder  pccc      N
##           <int> <int>  <int>
## 1:            0     0 457223
## 2:            1     0    615
## 3:            1     1  87738
mdcr_v_pccc[, .N, keyby = .(medicalcoder = any_transplant, pccc = transplant)]
## Key: <medicalcoder, pccc>
##    medicalcoder  pccc      N
##           <int> <int>  <int>
## 1:            0     0 525925
## 2:            1     0   4863
## 3:            1     1  14788
```

Several GitHub issues have been created for *pccc* to correct these issues for specific ICD codes:

- ICD-9 349.1
- ICD-9 V56
- ICD-10 Z49
- ICD-9 86.06
- ICD-9 V45.85
- ICD-9 V53.3

- ICD-9 V53.91
- ICD-9 V65.46
- ICD-9 37.52
- ICD-9 V42.0
- ICD-10 Z94

6.4 Summary of *medicalcoder* vs Other Implementations

Using the MIMIC-IV dataset, we compared encounter-level *medicalcoder* results with comparator implementations for shared algorithms and compatible inputs. The comparison focuses on whether outputs agree after harmonizing structural differences in returned objects and severity conventions.

Table 3: Summary of encounter-level agreement between *medicalcoder* and comparator implementations using MIMIC-IV v3.1 data, a total of 687,203 encounters.

medicalcoder method vs. Comparator	Discordant Encounters	Agreement summary	Main reason for discordance
`charlson_mimicivcode` vs MIMIC-IV Code	0	Exact match after harmonizing severity handling	Structural output/severity handling
`charlson_quan2011` vs *comorbidity*	0	Exact match for shared flags	Regex/prefix matching vs valid-code lookup
`elixhauser_quan2005` vs *comorbidity*	0	Exact match for shared flags	Extra medicalcoder summary/index fields
`pccc_v2.0` vs *pccc*	5,476 (0.80%)	High agreement, with documented differences	Technology dependence/transplant mapping

7 Precomputed vs Regular Expressions

By default, *medicalcoder* maps ICD codes to comorbidities using precomputed valid-code sets and database-style joins to lookup tables. *comorbidity*, and many other utilities, use regular expressions or partial string matching to make the maps. This can lead to problems. For example, the strings "C189N" and "C189NOTACODE" are not a valid ICD code, and will be flagged as a cancer comorbidity via `comorbidity::comorbidity()` but will not be flagged by `medicalcoder::comorbidities()`.

```r
DF <-
  data.frame(
    icd_codes = c("C1", "C18", "C189", "C189N", "C189NOTACODE"),
    codeids   = c("C1", "C18", "C189", "C189N", "C189NOTACODE"),
    stringsAsFactors = FALSE
  )
canc <-
  comorbidity::comorbidity(
    x = DF,
    id = "codeids",
    code = "icd_codes",
    map = "charlson_icd10_quan",
    assign0 = TRUE
  )[, c("codeids", "canc")]
mal <-
  medicalcoder::comorbidities(
    data = DF,
    id.var = "codeids",
    icd.codes = "icd_codes",
    method = "charlson_quan2005",
    poa = 1,
    primarydx = 0
    )[, c("codeids", "mal")]

merge(x = canc, mal, by = c("codeids"))
##        codeids canc mal
## 1           C1    0   0
## 2          C18    1   1
## 3         C189    1   1
## 4        C189N    1   0
## 5 C189NOTACODE    1   0
```

There are cases where a regular expressions could be useful. Consider the ICD-10-GM (German version of ICD-10). As of *medicalcoder* version 0.9.0, the ICD-10-GM codes are not part of the internal ICD database.

There are codes in ICD-10-GM which are not in the CDC, CMS, WHO, IHACPA, or Socialstyrelsen systems which are part of the *medicalcoder* ICD database. For example, ICD-10-GM E11.74: Type 2 diabetes mellitus With multiple complications With diabetic foot syndrome, controlled, and E11.75: Type 2 diabetes mellitus With multiple complications With diabetic foot syndrome, uncontrolled. These two codes are extensions under the E11 category; subcategory E11.7 which is listed in Quan et al. (2005) as diabetes with complications for Charlson and Elixhauser.

```r
subset(
  x = medicalcoder::get_icd_codes(with.description = TRUE),
```

```r
  subset = startsWith(code, "E117"),
  select = c("icdv", "dx", "full_code", "code", "src", "desc")
)
##        icdv dx full_code  code             src
## 154640   10  1     E11.7  E117 socialstyrelsen
## 154641   10  1     E11.7  E117             cdc
## 154642   10  1     E11.7  E117             who
## 154643   10  1     E11.7  E117          ihacpa
## 154644   10  1     E11.7  E117             who
## 154645   10  1    E11.70 E1170          ihacpa
## 154646   10  1    E11.71 E1171          ihacpa
## 154647   10  1    E11.72 E1172          ihacpa
## 154648   10  1    E11.73 E1173          ihacpa
##
## 154640                                              Diabetes mellitus typ 2 - med multipla komplika
## 154641                                 Non-insulin-dependent diabetes mellitus with multiple complic
## 154642                                 Non-insulin-dependent diabetes mellitus with multiple complic
## 154643                                               Type 2 diabetes mellitus with multiple complic
## 154644                                               Type 2 diabetes mellitus with multiple complic
## 154645   Non-insulin-dependent diabetes mellitus with multiple complications, not stated as uncont
## 154646 Type 2 diabetes mellitus with multiple microvascular or other specified nonvascular complic
## 154647                                         Type 2 diabetes mellitus with features of insulin resi
## 154648                                      Type 2 diabetes mellitus with foot ulcer due to multiple

subset(
  x = medicalcoder::get_charlson_codes(),
  subset = startsWith(code, "E117") & charlson_quan2005 == 1L,
  select = c("icdv", "dx", "full_code", "code", "condition")
)
##      icdv dx full_code  code condition
## 6019   10  1     E11.7  E117       dmc
## 6020   10  1    E11.70 E1170       dmc
## 6021   10  1    E11.71 E1171       dmc
## 6022   10  1    E11.72 E1172       dmc
## 6023   10  1    E11.73 E1173       dmc

subset(
  x = medicalcoder::get_elixhauser_codes(),
  subset = startsWith(code, "E117") & elixhauser_quan2005 == 1L,
  select = c("icdv", "dx", "full_code", "code", "condition")
)
##      icdv dx full_code  code condition
## 5554   10  1     E11.7  E117      DMCX
## 5555   10  1    E11.70 E1170      DMCX
## 5556   10  1    E11.71 E1171      DMCX
## 5557   10  1    E11.72 E1172      DMCX
## 5558   10  1    E11.73 E1173      DMCX
```

Thus, if you have a data-set with ICD-10-GM codes, you should expect false negative flags for diabetes with complications when using the default settings in `medicalcoder::comorbidities()`. In the following example we have E11.72, E11.73, E11.74, and E11.75. E11.72 and E11.73 should map to complex diabetes since those codes overlap with the IHACPA codes.

```
# data from https://gesund.bund.de/en/icd-code-suche/e11-7
# all of the description start with "Type 2 diabetes mellitus With
# multiple complications With" and the continue as:
#   E11.72: other multiple complications, controlled
#   E11.73: other multiple complications, uncontrolled
#   E11.74: diabetic foot syndrome, controlled
#   E11.75: diabetic foot syndrome, uncontrolled

GMdata <- data.table::fread(text ="
CodeID;ICD-Code
ICD-10-GM E11.72;E11.72
ICD-10-GM E11.73;E11.73
ICD-10-GM E11.74;E11.74
ICD-10-GM E11.75;E11.75"
)

common_args <-
  list(
    data      = GMdata,
    id.vars   = "CodeID",
    icd.codes = "ICD-Code",
    dx        = 1,
    icdv      = 10,
    poa       = 1,
    primarydx = 0
  )

do.call(
  what = medicalcoder::comorbidities,
  args = c(common_args, list(method = "charlson_quan2005"))
)[, c("CodeID", "dm", "dmc")]
##
## Comorbidities via charlson_quan2005
##
##              CodeID    dm   dmc
##              <char> <int> <int>
## 1: ICD-10-GM E11.72     0     1
## 2: ICD-10-GM E11.73     0     1
## 3: ICD-10-GM E11.74     0     0
## 4: ICD-10-GM E11.75     0     0

do.call(
  what = medicalcoder::comorbidities,
```

```r
  args = c(common_args, list(method = "elixhauser_quan2005"))
)[, c("CodeID", "DM", "DMCX")]
##
## Comorbidities via elixhauser_quan2005
##
##                CodeID    DM  DMCX
##                <char> <int> <int>
## 1: ICD-10-GM E11.72     0     1
## 2: ICD-10-GM E11.73     0     1
## 3: ICD-10-GM E11.74     0     0
## 4: ICD-10-GM E11.75     0     0
```

If, however, you use `mapping = "regex"` in the call to `medicalcoder::comorbidities()` (new in v0.9.0), then these conditions will be flagged, at least for Charlson, the Elixhauser regex has not yet been implemented.

```r
do.call(
  what = medicalcoder::comorbidities,
  args = c(common_args, list(method = "charlson_quan2005", mapping = "regex"))
)[, c("CodeID", "dm", "dmc")]
##
## Comorbidities via charlson_quan2005
##
##                CodeID    dm   dmc
##                <char> <int> <int>
## 1: ICD-10-GM E11.72     0     1
## 2: ICD-10-GM E11.73     0     1
## 3: ICD-10-GM E11.74     0     1
## 4: ICD-10-GM E11.75     0     1

try(
  do.call(
    what = medicalcoder::comorbidities,
    args = c(common_args, list(method = "elixhauser_quan2005", mapping = "regex"))
  )[, c("CodeID", "DM", "DMCX")]
, silent = TRUE) |>
print()
## [1] "Error : mapping = \"regex\" for Elixhauser methods has not yet been implemented\n"
## attr(,"class")
## [1] "try-error"
## attr(,"condition")
## <simpleError: mapping = "regex" for Elixhauser methods has not yet been implemented>
```

The current regular expressions are available to end users via

```r
str(medicalcoder:::..mdcr_internal_charlson_regex..)
## 'data.frame':	117 obs. of  11 variables:
## $ condition : chr "aids" "aids" "aidshiv" "aidshiv" ...
## $ icdv : int 9 10 9 10 10 10 9 9 10 10 ...
## $ dx : int 1 1 1 1 1 1 1 1 1 1 ...
## $ pattern : chr
##    "^(0(0(3\\.?1|7\\.?[24])|1[0-8]|31|46\\.?3|54|78\\.?5)|1(1([245]|7\\.?5)|3"..
##    __truncated__
##    "^(A(0(2\\.?1|7\\.?[23])|1[5-9]|31|81\\.?2)|B(00|25|3[7-9]|45|5[89])|C(46|"..
##    "^04[2-4]" "^(B2[0-4]|F02\\.?4|O98\\.?7|R75\\.?|Z(21\\.?9|71\\.?7))" ...
## $ charlson_cdmf2019 : int 1 1 0 0 0 0 1 0 0 0 ...
## $ charlson_deyo1992 : int 0 0 1 0 0 0 0 1 0 0 ...
## $ charlson_ludvigsson2021 : int 0 0 0 1 0 0 0 0 0 1 ...
## $ charlson_mimicivcode : int 0 0 1 0 1 0 1 0 0 0 ...
## $ charlson_quan2005 : int 0 0 1 0 1 0 1 0 0 0 ...
## $ charlson_sundararajan2004: int 0 0 0 0 0 1 0 0 1 0 ...
## $ charlson_quan2011 : int 0 0 1 0 1 0 1 0 0 0 ...
```

The regular expression patterns have been written to match both full and compact ICD codes.

Revisiting the first example in this section, we can see that using `mapping = "regex"` in `medicalcoder::comorbidities()` will give the same false-positives as `comorbidity::comorbidity()` does. The utility exists, but it should be used with care.

```r
mal2 <-
  medicalcoder::comorbidities(
    data = DF,
    id.var = "codeids",
    icd.codes = "icd_codes",
    method = "charlson_quan2005",
    poa = 1,
    primarydx = 0,
    mapping = "regex"
    )[, c("codeids", "mal")]

merge(
  x = merge(x = canc, mal, by = c("codeids")),
  y = mal2,
  by = c("codeids"),
  suffixes = c("_precomputed", "_by_regex")
)
##        codeids canc mal_precomputed mal_by_regex
## 1           C1    0               0            0
## 2          C18    1               1            1
## 3         C189    1               1            1
## 4        C189N    1               0            1
## 5 C189NOTACODE    1               0            1
```

As of *medicalcoder* 0.9.0 several Charlson methods have exposed regular expressions for mapping ICD codes to conditions. Future releases of *medicalcoder* will extend this to Elixhauser and PCCC methods.

8 Longitudinal Flagging

Under the assumption that all relevant ICD codes are (re)reported on each encounter then there is no need to carry-forward comorbidity flags. However, that assumption is rarely valid. *medicalcoder* provides a utility for indefinite carry-forward of a condition. As reported in the primary manuscript this includes accounting for conditions that should not be reported on a specific encounter but are reported on the following encounters. In the following we will explore some of the pros and cons of the indefinite carry-forward of comorbidities.

To simplify the work we will reapply the comorbidity algorithms using `enc_seq` instead of `hadm_id` so that the encounters are considered in chronologic order. We present here the code for Charlson, Elixhauser, and PCCC, which will be referenced several times within the remainder of this document.

```
medicalcoder_charlson_current <-
  medicalcoder::comorbidities(
    data        = mimicivDT,
    icd.codes   = "icd_code",
    id.vars     = c("subject_id", "enc_seq"),
    icdv.var    = "icd_version",
    dx.var      = "dx",
    poa         = 1L,
    primarydx   = 0L,
    method      = "charlson_quan2011",
    flag.method = "current"
  )
```

```
medicalcoder_charlson_cumulative <-
  medicalcoder::comorbidities(
    data        = mimicivDT,
    icd.codes   = "icd_code",
    id.vars     = c("subject_id", "enc_seq"),
    icdv.var    = "icd_version",
    dx.var      = "dx",
    poa         = 1L,
    primarydx   = 0L,
    method      = "charlson_quan2011",
    flag.method = "cumulative"
  )
```

```r
medicalcoder_elixhauser_current <-
  medicalcoder::comorbidities(
    data        = mimicivDT,
    icd.codes   = "icd_code",
    id.vars     = c("subject_id", "enc_seq"),
    icdv.var    = "icd_version",
    dx.var      = "dx",
    poa         = 1L,
    primarydx   = 0L,
    method      = "elixhauser_quan2005",
    flag.method = "current"
  )
```

```r
medicalcoder_elixhauser_cumulative <-
  medicalcoder::comorbidities(
    data        = mimicivDT,
    icd.codes   = "icd_code",
    id.vars     = c("subject_id", "enc_seq"),
    icdv.var    = "icd_version",
    dx.var      = "dx",
    poa         = 1L,
    primarydx   = 0L,
    method      = "elixhauser_quan2005",
    flag.method = "cumulative"
  )
```

```r
medicalcoder_pcccv2.0_current <-
  medicalcoder::comorbidities(
    data        = mimicivDT,
    icd.codes   = "icd_code",
    id.vars     = c("subject_id", "enc_seq"),
    icdv.var    = "icd_version",
    dx.var      = "dx",
    poa         = 1L,
    method      = "pccc_v2.0",
    flag.method = "current"
  )
```

```r
medicalcoder_pcccv2.0_cumulative <-
  medicalcoder::comorbidities(
    data        = mimicivDT,
    icd.codes   = "icd_code",
    id.vars     = c("subject_id", "enc_seq"),
    icdv.var    = "icd_version",
    dx.var      = "dx",
    poa         = 1L,
    method      = "pccc_v2.0",
```

```
    flag.method = "cumulative"
  )
```

```
medicalcoder_pcccv3.1_current <-
  medicalcoder::comorbidities(
    data        = mimicivDT,
    icd.codes   = "icd_code",
    id.vars     = c("subject_id", "enc_seq"),
    icdv.var    = "icd_version",
    dx.var      = "dx",
    poa         = 1L,
    method      = "pccc_v3.1",
    flag.method = "current"
  )
```

```
medicalcoder_pcccv3.1_cumulative <-
  medicalcoder::comorbidities(
    data        = mimicivDT,
    icd.codes   = "icd_code",
    id.vars     = c("subject_id", "enc_seq"),
    icdv.var    = "icd_version",
    dx.var      = "dx",
    poa         = 1L,
    method      = "pccc_v3.1",
    flag.method = "cumulative"
  )
```

8.1 Severity of Disease

Let's look at the impact of longitudinal flagging vs encounter level flagging of Charlson comorbidities.

Figure 1 shows the number of encounters flagged with a given condition for `flag.method = 'current'` and `flag.method = 'cumulative'` for the Charlson condition flagged by `medicalcoder::comorbidities()`. For most conditions the number of encounters flagged is greater when using the cumulative flag method compared to the current flag method. However, there is a notable counter example: dm (diabetes without complications) and dmc (diabetes with complications). *medicalcoder* will carry forward the severity of disease and report the more severe version after it has been reported.

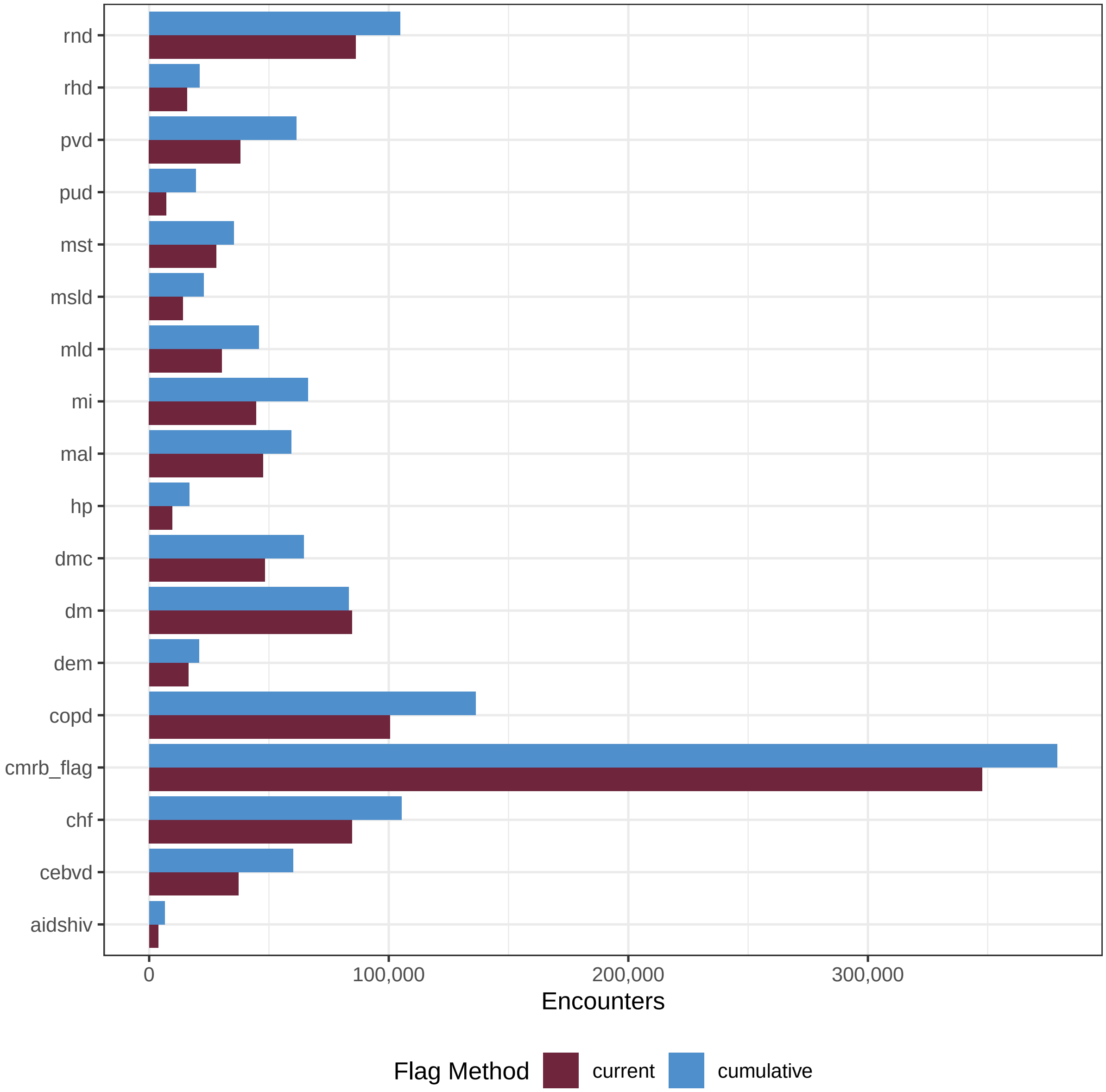

Figure 1: Number of encounters flagged with Charlson comorbidities based on encounter only (`flag.method = "current"`) versus the whole patient history (`flag.method = "cumulative"`).

An example of a patient record where on an encounter level flagging approach diabetes will be missed entirely and the severity is inaccurate is in Table 4. Subject 10009326 had 17 encounters. At least one ICD code mapping to uncomplicated diabetes under the Charlson *a la* Quan et al. (2005) appeared in 12 encounters, and 1 encounter for complicated diabetes. Under an encounter-level only flagging of comorbidities, this patient would erroneously not have diabetes of any

kind on 4 encounters. Using the longitudinal flagging in `medicalcoder::comorbidities()` via `flag.method = 'cumulative'`, we have diabetes flagged on all encounters, and the severity correctly marked for encounters later in the patient record.

Table 4: Example impact of flagging Diabetes using encounter-level data only vs a longitudinal approach. These are the Charlson comorbidities for MIMIC-IV subject_id 10009326 using `medicalcoder::comorbidities()` with `method = "charlson_quan2011"`, `flag.method = "current"` and `flag.method = "cumulative"`.

	Current		Cumulative	
Encounter	Complicated	Uncomplicated	Complicated	Uncomplicated
1		X		X
2				X
3		X		X
4		X		X
5				X
6		X		X
7		X		X
8		X		X
9				X
10		X		X
11				X
12	X		X	
13		X	X	
14		X	X	
15		X	X	
16		X	X	
17		X	X	

8.1.1 Elixhauser

For Elixhauser, Figure 2, shows similar behavior for diabetes as Charlson.

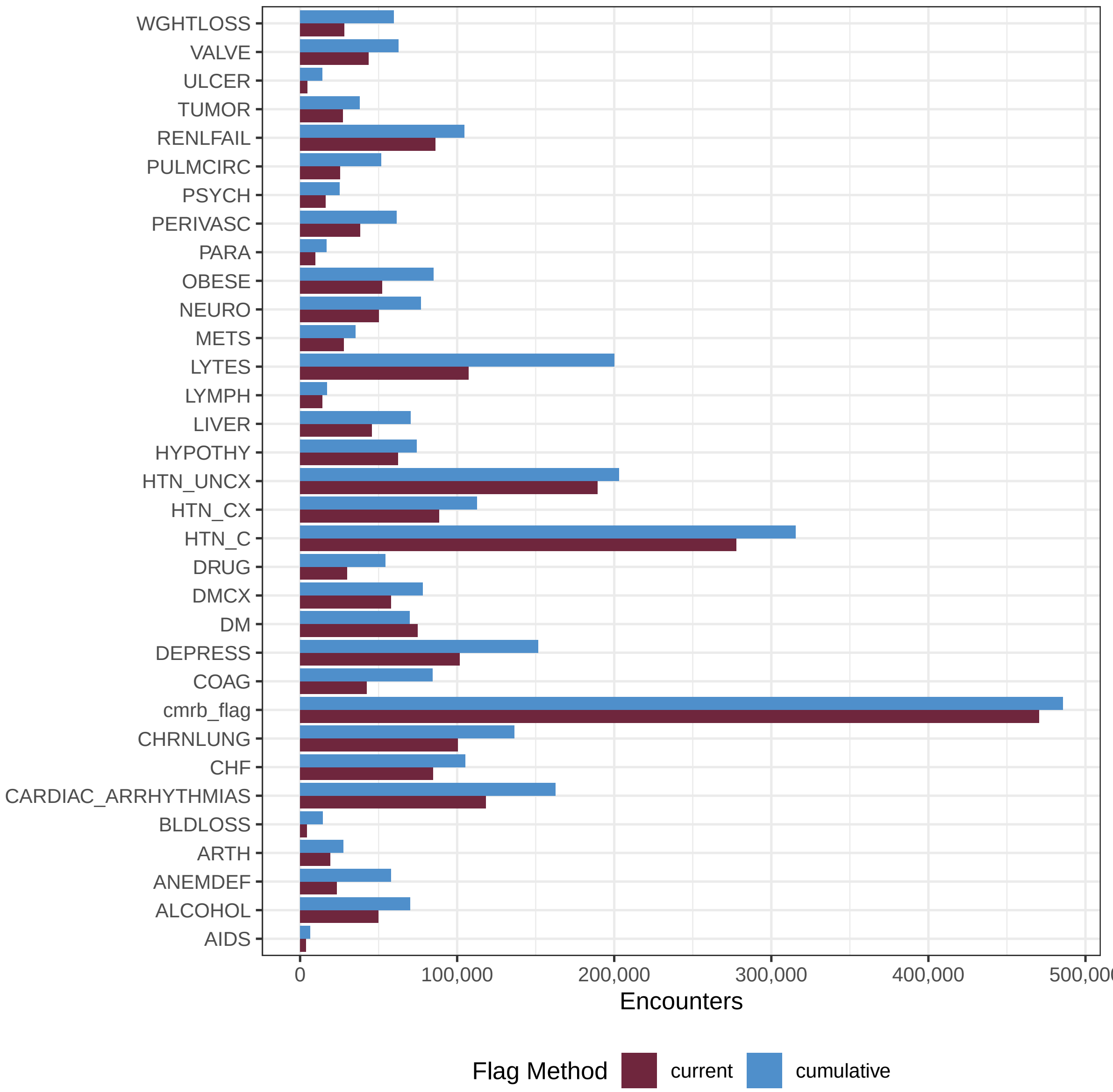

Figure 2: Number of encounters flagged with Elixhauser comorbidities based on encounter only (`flag.method = current`) versus the whole patient history (`flag.method = "cumulative"`).

8.1.2 PCCC

Figure 3 and Figure 4 illustrate the impact of the flagging method when identifying PCCC comorbidities under PCCC v2 and PCCC v3 respectively. Figure 4 is of particular interest as

the way technology-dependent codes in PCCC v3 are handled has considerable impact on the flagged comorbidities.

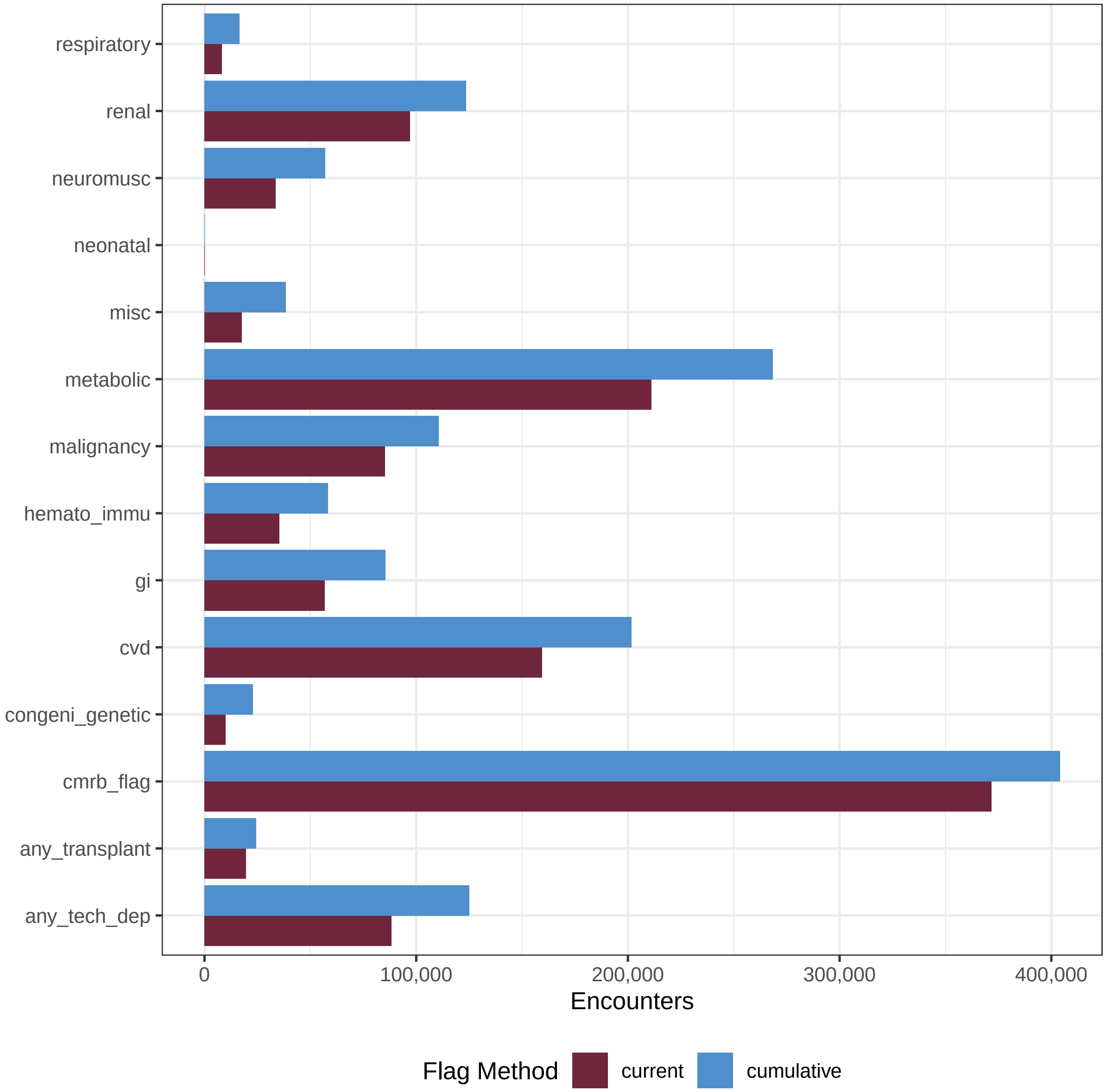

Figure 3: Number of encounters flagged with PCCC v2 comorbidities based on encounter only (`flag.method = current`) versus the whole patient history (`flag.method = "cumulative"`).

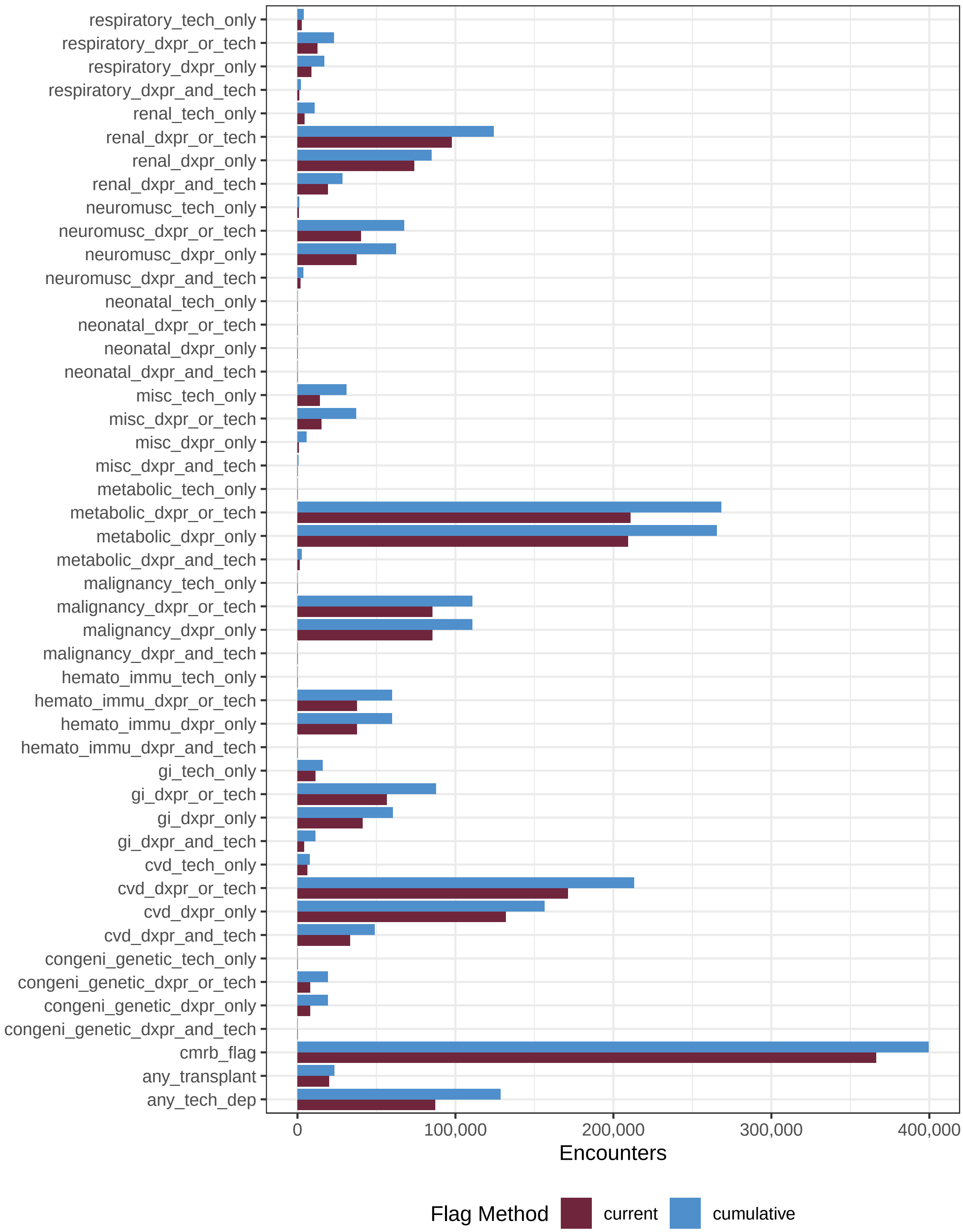

Figure 4: Number of encounters flagged with PCCC v3 comorbidities based on encounter only (`flag.method = current`) versus the whole patient history (`flag.method = "cumulative"`).

Let's look at the records for `subject_id = 10728333`. Table 5 shows the flagging of metabolic, miscellaneous, and respiratory conditions for PCCC v3 using `flag.method = 'current'` or `flag.method = 'cumulative'`. For this patient, on encounter 1, a technology-dependent ICD code related to a respiratory comorbidity was observed in the patient record. No other ICD codes related to a PCCC v3 comorbidity were observed for encounter 1. As such, no comorbidities are flagged for encounter 1 as at least one non-technology-dependent ICD code is required for any comorbidity to be flagged. Encounter 2 had no PCCC v3 related ICD codes reported. Encounter 3 had a technology-dependent ICD code for a miscellaneous comorbidity reported and again, because no non-technology-dependent ICD was reported no comorbidity is flagged. Starting on encounter 4, a non-technology-dependent ICD code for a metabolic comorbidity was reported. Under the `flag.method = 'current'` that is the only code that is reported and thus only one comorbidity is reported. Under `flag.method = 'cumulative'` the technology-dependent ICD codes from encounters 1 and 3 have been carried forward in the record to encounter 4 and are thus flagged. If the end user were to try to build the cumulative comorbidities from the results of the `flag.method = 'current'` results, the end user would never capture the miscellaneous comorbidity, and would fail to capture the respiratory comorbidity until encounter 6, and would incorrectly classify as non-technology-dependent until the final encounter.

Table 5: Comparison of encounter-level and cumulative flagging of PCCC v3 conditions for MIMIC-IV subject 10728333. Cells marked with DxPr denote that the comorbidity was flagged due to an ICD code that is not technology-dependent. Tech denotes flagging of a comorbidity based on the presence of at least one technology-dependent ICD code and the presence of at least one non-technology-dependent code for another comorbidity. DxPrTech denotes flagging due to both technology-dependent and non-technology-dependent ICD codes. Under the Any Tech columns we mark the encounters where some tech dependence is identified. For this patient, on encounter 1, a technology-dependent ICD code for a respiratory comorbidity was found in the subject's record. Because no non-technology-dependent code was observed on encounter 1, under PCCC v3 this subject has no comorbidities for encounter 1. This persists for encounters 2 and 3 with respect to respiratory and on encounter 3 for miscellaneous as well. On encounter 4 a non-technology-dependent code for a metabolic comorbidity was reported. Under a cumulative flagging paradigm, the miscellaneous and respiratory comorbidities are also flagged as the reported codes from encounters 3 and 1 have been carried forward respectively. Under the 'current' flagging method miscellaneous is never flagged as the technology-dependent code never occurs on the same encounter as the non-technology-dependent code.

	Metabolic		Miscellaneous		Respiratory		Any Tech		Number of Comorbidities	
Encounter	Current	Cumulative	Current	Cumulative	Current	Cumulative	Current	Cumulative	Current	Cumulative
1					*	*			0	0
2						†			0	0
3			*	*		†			0	0
4	DxPr	DxPr		Tech		Tech		1	1	3
5	DxPr	DxPr		Tech		Tech		1	1	3
6	DxPr	DxPr		Tech	DxPr	DxPrTech		1	2	3
7	DxPr	DxPr		Tech		DxPrTech		1	1	3
8	DxPr	DxPr		Tech		DxPrTech		1	1	3
9	DxPr	DxPr		Tech		DxPrTech		1	1	3
10	DxPr	DxPr		Tech		DxPrTech		1	1	3
11		DxPr		Tech		DxPrTech		1	0	3
12		DxPr		Tech		DxPrTech		1	0	3
13	DxPr	DxPr		Tech	Tech	DxPrTech	1	1	2	3

* A technology-dependent code was reported on this encounter. Since no non-technology-dependent was reported no comorbidities are flagged.
† A technology-dependent code has been carried forward in the record. Since no non-technology-dependent was reported on this, or a prior, enconter, no comorbidities are flagged.

8.2 Diabetes - Chronic vs Acute Disease

Non-gestational diabetes is generally considered a chronic condition. Even when well managed, it is still a relevant diagnosis. The ICD-10 code defining diabetes (with and without complications) for Charlson comorbidities as defined by Quan et al. (2005, 2011), are all in the E10, E11, E12, E13, and E14 categories.

```
subset(
  x = medicalcoder::get_charlson_codes(),
  subset = charlson_quan2005 == 1L & condition %in% c("dm", "dmc") & icdv == 10,
)[["code"]] |>
substring(1, 3) |>
unique() |>
sort()
## [1] "E10" "E11" "E12" "E13" "E14"
```

Let's look at the records for those who have at least two encounters and at least one of the encounters was flagged with diabetes (with or without complications).

```
sids <-
  medicalcoder_charlson_current[
    ,
    .(encounters = length(unique(enc_seq)), any_dm = sum(dm + dmc)),
    by = .(subject_id)
  ][
    encounters > 1 & any_dm > 0,
    subject_id
  ]

dms <-
  merge(
    x = medicalcoder_charlson_current[
          subject_id %in% sids,
          .(subject_id, enc_seq, dm_current = dm, dmc_current = dmc)
        ],
    y = medicalcoder_charlson_cumulative[
          subject_id %in% sids,
          .(subject_id, enc_seq, dm_cumulative = dm, dmc_cumulative = dmc)
        ],
    all = TRUE,
    by = c("subject_id", "enc_seq")
  )
data.table::setDT(dms)
dms[, current := (dm_current + dmc_current)]
dms[, cumulative := (dm_cumulative + dmc_cumulative)]
dms[, total_encs := max(enc_seq), by = .(subject_id)]
dms
## Key: <subject_id, enc_seq>
##          subject_id enc_seq dm_current dmc_current dm_cumulative dmc_cumulative
##               <int>   <int>      <int>       <int>         <int>          <int>
##       1:   10000635       1          1           0             1              0
##       2:   10000635       2          1           0             1              0
##       3:   10000980       1          1           0             1              0
##       4:   10000980       2          1           0             1              0
```

```
##      5:   10000980       3          1           0             1             0
##     ---
## 146739:   19999287       3          1           0             1             0
## 146740:   19999379       1          1           0             1             0
## 146741:   19999379       2          1           0             1             0
## 146742:   19999828       1          1           0             1             0
## 146743:   19999828       2          1           0             1             0
##         current cumulative total_encs
##           <int>      <int>      <int>
##      1:       1          1          2
##      2:       1          1          2
##      3:       1          1          7
##      4:       1          1          7
##      5:       1          1          7
##     ---
## 146739:       1          1          3
## 146740:       1          1          2
## 146741:       1          1          2
## 146742:       1          1          2
## 146743:       1          1          2
```

We count cumulative flags on encounters where DM (with or without complications) is flagged on both a preceding and a following encounter, or only after a preceding encounter.

```
dms[
  ,
  current_flags_before := as.integer((cumsum(current) - current) > 0),
  by = .(subject_id)
]

dms[
  ,
  current_flags_after := as.integer(rev(cumsum(rev(current)) - current) > 0),
  by = .(subject_id)
]

dms[
  ,
  cumulative_between_current_flags :=
    as.integer(
      (current != cumulative) &
      (current_flags_before == 1L) &
      (current_flags_after == 1L)
    )
]

dms[
```

```
  ,
  cumulative_only_after_current_flag :=
    as.integer(
      (current != cumulative) &
      (current_flags_before == 1L) &
      (current_flags_after == 0L)
    )
]

# Number of Encounters:
dms[
  ,
  .(Encounters = .N),
  keyby = .(cumulative_between_current_flags, cumulative_only_after_current_flag)
]
## Key: <cumulative_between_current_flags, cumulative_only_after_current_flag>
##    cumulative_between_current_flags cumulative_only_after_current_flag
##                               <int>                              <int>
## 1:                               0                                  0
## 2:                               0                                  1
## 3:                               1                                  0
##    Encounters
##         <int>
## 1:     131749
## 2:       9219
## 3:       5775
```

At a subject-level, the

```
# counts of subjects with encounters flagged
dms[
    ,
    .(
      between = any(cumulative_between_current_flags),
      after = any(cumulative_only_after_current_flag)
    ),
    by = .(subject_id)
  ][
    ,
    .(Subjects = .N),
    keyby = .(between, after)
  ]
## Key: <between, after>
##    between  after Subjects
##     <lgcl> <lgcl>    <int>
## 1:   FALSE  FALSE    21241
## 2:   FALSE   TRUE     3267
```

```
## 3:     TRUE  FALSE      2086
## 4:     TRUE   TRUE       462
```

Longitudinal flagging of a chronic condition could be beneficial, especially in cases where we see the condition flagged on encounters preceding and following a specific encounter.

End users are cautioned to understand the specific conditions and comorbidity methods being used before applying the indefinite carry-forward. For example, gestational diabetes is part of the comorbidity mappings for the AHRQ Elixhauser variants based on ICD-10 codes. Note that ICD-10 category E08, E09, E10, E11, E13, *and* O24 are all diabetes codes with O24 codes mapping to uncomplicated diabetes.

```
ahrq_icd10_dm_codes <-
  subset(
    x = medicalcoder::get_elixhauser_codes(),
    subset = elixhauser_ahrq_icd10 == 1 & condition %in% c("DIAB_CX", "DIAB_UNCX"),
    select = c("code", "condition")
  )
data.table::setDT(ahrq_icd10_dm_codes)
ahrq_icd10_dm_codes[, category := substring(code, 1, 3)]

ahrq_icd10_dm_codes[, .N, keyby = .(category, condition)] |>
  data.table::dcast(category ~ condition, value.var = "N", fill = 0L)
## Key: <category>
##    category DIAB_CX DIAB_UNCX
##      <char>   <int>     <int>
## 1:      E08      89         5
## 2:      E09      89         5
## 3:      E10      89         3
## 4:      E11      89         6
## 5:      E13      89         5
## 6:      O24       0        42
```

In the MIMIC-IV data, there are subjects with both gestational and non-gestational diabetes, subjects with only gestational diabetes, and subjects with only non-gestational diabetes.

```
DT <- mimicivDT[icd_code %in% ahrq_icd10_dm_codes[condition == "DIAB_UNCX", code]]
DT[, Gestational := any(startsWith(icd_code, "O")), by = .(subject_id)]
DT[, `Non-Gestational` := any(startsWith(icd_code, "E")), by = .(subject_id)]
DT[
  , unique(.SD), .SDcols = c("subject_id", "Gestational", "Non-Gestational")
  ][
  , .(Subjects = .N), keyby = .(Gestational, `Non-Gestational`)
  ]
## Key: <Gestational, Non-Gestational>
```

```
##    Gestational Non-Gestational Subjects
##         <lgcl>          <lgcl>    <int>
## 1:       FALSE            TRUE    16785
## 2:        TRUE           FALSE      725
## 3:        TRUE            TRUE      139
```

Since the AHRQ variant of Elixhauser comorbidities includes both chronic and non-chronic versions of diabetes, `flag.method = "cumulative"` may result in false positives of diabetes.

8.3 Metastatic Tumors

Metastatic tumors could be considered non-chronic conditions as the tumors could be removed via surgery or treated with other therapies. As such, the flags reported via `flag.method = "cumulative"` for malignancy (Charlson and PCCC), or solid tumors (Elixhauser) should not be interpreted as "present disease" but as "a history of *observed* disease."

We empathize *observed disease* because there are ICD codes specifically for personal history of malignancies. We collect those ICD codes and the codes mapping to malignancies per Quan et al. (2005, 2011) and subset the `mimicivDT` to subjects with these codes.

```
personal_history_of_malignant <-
  subset(
    x = medicalcoder::get_icd_codes(with.description = TRUE),
    subset = src == "cms" & grepl("personal history of malignant", desc, ignore.case = TRUE),
    select = c("code", "icdv", "dx")
  )
personal_history_of_malignant[["history_of"]] <- 1L

charlson_quan2011_mal <-
  subset(
    x = medicalcoder::get_charlson_codes(),
    subset = condition == "mal" & charlson_quan2011 == 1L,
    select = c("code", "icdv", "dx")
  )
charlson_quan2011_mal[["observed_mal"]] <- 1L

mal <-
  merge(
    x = mimicivDT[!is.na(icd_code), .(subject_id, enc_seq, icd_code, icd_version, dx)],
    y = personal_history_of_malignant,
    all.x = TRUE,
    by.x = c("icd_code", "icd_version", "dx"),
    by.y = c("code", "icdv", "dx")
  )
```

```r
mal <-
  merge(
    x = mal,
    y = charlson_quan2011_mal,
    all.x = TRUE,
    by.x = c("icd_code", "icd_version", "dx"),
    by.y = c("code", "icdv", "dx")
  )
data.table::setkey(mal, subject_id, enc_seq)

# set flag for any history or observation of a malignancy
mal <-
  mal[
    ,
    .(
      enc_history_of   = as.integer(any(history_of, na.rm = TRUE)),
      enc_observed_mal = as.integer(any(observed_mal, na.rm = TRUE))
    ),
    by = .(subject_id, enc_seq)
  ]
mal[, subject_history_of   := as.integer(sum(enc_history_of) > 0), by = .(subject_id)]
mal[, subject_observed_mal := as.integer(sum(enc_observed_mal) > 0), by = .(subject_id)]

mal <- mal[subject_history_of + subject_observed_mal > 0]

# join on the cumulative flags from medicalcoder for both malignancy and
# metastatic solid tumors.  An encounter with both a malignancy and a metastatic
# solid tumor will have the malignancy flag set to zero and retain only the more
# severe condition.
mal <-
  merge(
    x = mal,
    y = medicalcoder_charlson_cumulative[
          , .(subject_id, enc_seq, mdcr_cummulative_mal = mal, mdcr_cummulative_mst = mst)
        ],
    all.x = TRUE,
    by = c("subject_id", "enc_seq")
  )

mal[, .(Encounters = .N), keyby = .(enc_observed_mal, enc_history_of)]
## Key: <enc_observed_mal, enc_history_of>
##    enc_observed_mal enc_history_of Encounters
##               <int>          <int>      <int>
## 1:                0              0      40443
## 2:                0              1      47083
## 3:                1              0      58910
## 4:                1              1       8222
```

The next chunk will count the number of subjects with reported history of a malignancy and those with an observed malignancy

```
subject_history <-
  mal[
    ,
    lapply(lapply(.SD, any), as.integer),
    .SDcols = c("subject_history_of", "subject_observed_mal"),
    by = .(subject_id)
  ]
subject_history[
    ,
    .(subjects = .N), keyby = .(subject_history_of, subject_observed_mal)
  ]
## Key: <subject_history_of, subject_observed_mal>
##    subject_history_of subject_observed_mal subjects
##                 <int>                <int>    <int>
## 1:                  0                    1    18127
## 2:                  1                    0    15229
## 3:                  1                    1     9360
```

What is notable in the above is that there are 15,229 (35.65%) patients who have only a personal history of malignancy in the dataset. If the cumulative flag was to be considered a "history of", these patients would be considered false negatives. The `flag.method = 'cumulative'` can only be interpreted as "current or history of observed disease."

8.4 AHRQ (2022+) Example - POA

AHRQ Elixhauser for years 2022 and beyond use ICD-10 diagnostic codes and require some ICD codes to be present-on-admission for the comorbidity to be flagged. The MIMIC data does not have a present-on-admission flag. For this example let's assume that all codes are not POA.

```
common_args <-
  list(
    data = mimicivDT,
    id.vars = c("subject_id", "enc_seq"),
    icd.codes = "icd_code",
    icdv.var = "icd_version",
    dx.var = "dx",
    poa = 0L,
    primarydx = 0L,
    method = "elixhauser_ahrq_icd10"
  )
```

```r
flgcurrent <-
  do.call(
    medicalcoder::comorbidities,
    c(common_args, list(flag.method = "current"))
  )

flgcumulative <-
  do.call(
    medicalcoder::comorbidities,
    c(common_args, list(flag.method = "cumulative"))
  )
```

Let's look at subject_id 19997538. This subject has ICD code K76.0 reported on encounter 1.

```r
mimicivDT[subject_id == 19997538 & icd_code == "K760"]
## Key: <subject_id, enc_seq, hadm_id>
##    subject_id  hadm_id    dx seq_num icd_code icd_version chartdate      age
##         <int>    <int> <int>   <int>   <char>       <int>    <IDat>    <num>
## 1:   19997538 22701415     1      15     K760          10      <NA> 53.33048
##     admittime enc_seq                cid
##        <POSc>   <int>             <char>
## 1: 2168-05-01       1 19997538__22701415
```

K76.0 maps to mild liver disease and is required to be POA for the condition to be flagged.

```r
subset(medicalcoder::get_icd_codes(with.description = TRUE), full_code == "K76.0")
##        icdv dx full_code code             src known_start known_end
## 177671   10  1     K76.0 K760             cms        2014      2026
## 177672   10  1     K76.0 K760             cdc        2001      2025
## 177673   10  1     K76.0 K760          ihacpa        2020      2026
## 177674   10  1     K76.0 K760             who        2008      2021
## 177675   10  1     K76.0 K760 socialstyrelsen        1997      2026
##        assignable_start assignable_end
## 177671             2014           2026
## 177672             2001           2025
## 177673             2020           2026
## 177674             2008           2021
## 177675             1997           2026
##                                                desc desc_start desc_end
## 177671 Fatty (change of) liver, not elsewhere classified       2014     2026
## 177672 Fatty (change of) liver, not elsewhere classified       2001     2025
## 177673 Fatty (change of) liver, not elsewhere classified       2020     2026
## 177674 Fatty (change of) liver, not elsewhere classified       2008     2021
## 177675     Fettlever som ej klassificeras på annan plats       1997     2026
subset(medicalcoder::get_elixhauser_codes(), full_code == "K76.0")
```

```
##      icdv dx full_code code poaexempt condition elixhauser_ahrq_web
## 9300   10  1     K76.0 K760        NA     LIVER                   0
## 9301   10  1     K76.0 K760         0 LIVER_MLD                  NA
##      elixhauser_elixhauser1988 elixhauser_quan2005 elixhauser_ahrq2022
## 9300                         0                   1                  NA
## 9301                        NA                  NA                   1
##      elixhauser_ahrq2023 elixhauser_ahrq2024 elixhauser_ahrq2025
## 9300                  NA                  NA                  NA
## 9301                   1                   1                   1
##      elixhauser_ahrq2026 elixhauser_ahrq_icd10
## 9300                  NA                    NA
## 9301                   1                     1
subset(medicalcoder::get_elixhauser_poa(), condition == "LIVER_MLD")
##    condition poa_required elixhauser_ahrq2022 elixhauser_ahrq2023
## 21 LIVER_MLD            1                   1                   1
##    elixhauser_ahrq2024 elixhauser_ahrq2025 elixhauser_ahrq2026
## 21                   1                   1                   1
##    elixhauser_ahrq_icd10
## 21                     1
```

When `flag.method = "current"` we see what we expect, mild liver disease is not flagged on any encounter. When `flag.method = "cumulative"` we see that on encounter 1 the condition is not flagged, as expected since it is not POA, but it is flagged on encounters 2 and 3 as the patient history is accounted for.

```
merge(
  x = flgcurrent,
  y = flgcumulative,
  by = c("subject_id", "enc_seq"),
  suffixes = c("_current", "_cumulative")
)[
  subject_id == 19997538,
  .SD,
  .SDcols = patterns("^(subject_id|enc_seq|LIVER_MLD_c)")
]
## Key: <subject_id, enc_seq>
##    subject_id enc_seq LIVER_MLD_current LIVER_MLD_cumulative
##         <int>   <int>             <int>                <int>
## 1:   19997538       1                 0                    0
## 2:   19997538       2                 0                    1
## 3:   19997538       3                 0                    1
```

9 Time Required To Compute

The times required (median and IQR) to apply the different comorbidity algorithms to the MIMIC-IV data are shown in Table 6. To process the 7,365,770 total rows of data for 364,627 unique subjects and a total of 687,203 unique hospital encounters, `medicalcoder::comorbidities()` is considerably faster than all the alternatives.

For this work we will include several additional calls to `medicalcoder::comorbidities()`.

The benchmarking is done using *microbenchmark* (Mersmann 2024) and calling each expression ten times.

Figure 5 shows the median time in seconds along with error bars for the IQR for the ten iterations. The reason for the drastic speed improvement between *medicalcoder* and other utilities is that *medicalcoder* uses precomputed maps between known ICD codes and conditions whereas *comorbidity* uses regular expressions, and *pccc* uses partial string matching. The MIMIC-IV code also uses partial string matching and lexicographical between statements. The numeric values represented in Figure 5 are reported in Table 6.

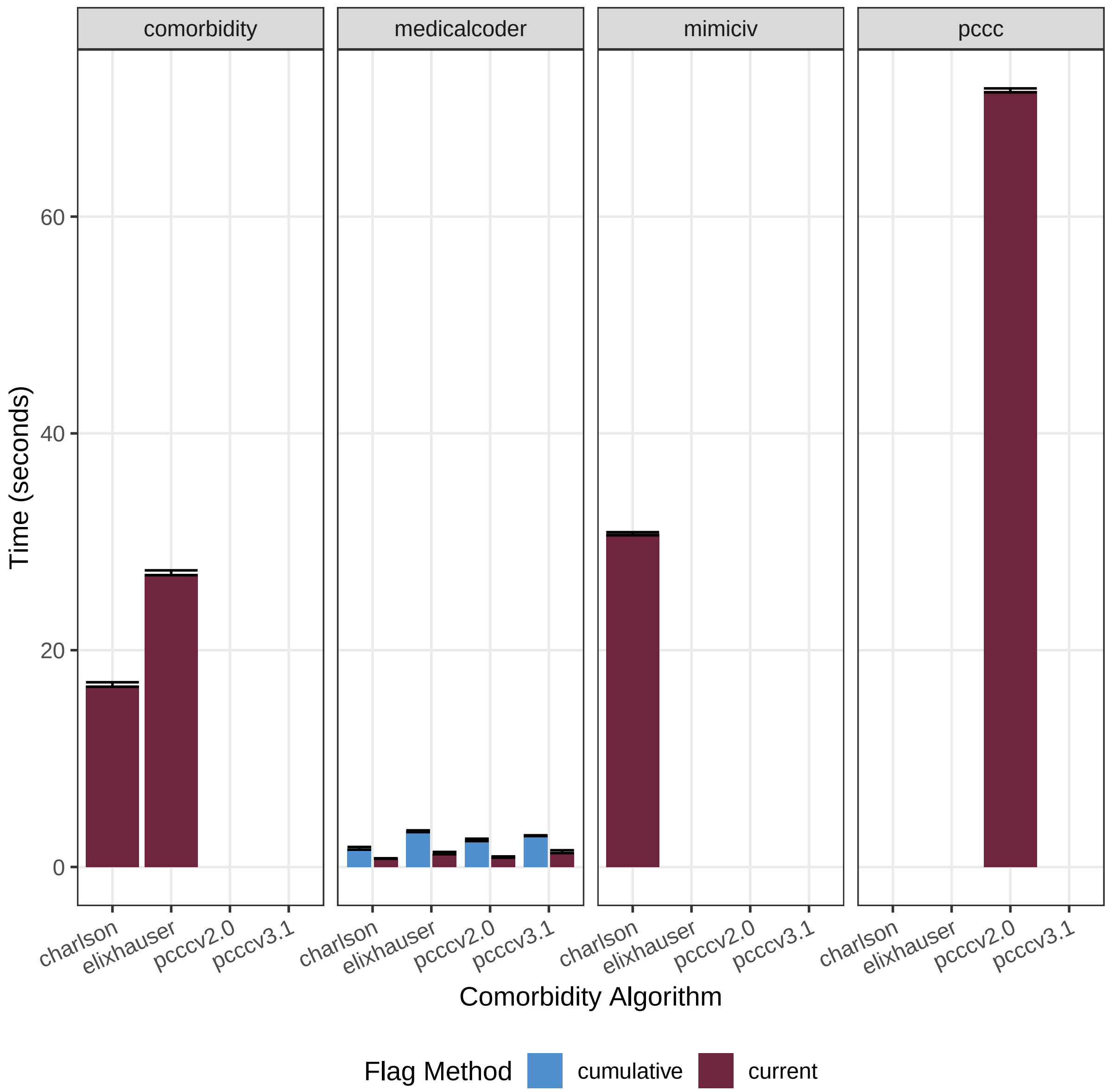

Figure 5: Time (height = median, error bars = IQR) in seconds to apply several comorbidity algorithms to the MIMIC-IV dataset. Each method was evaluated 10 times. See Table 8 for computational platform details.

Table 6: Time, in seconds, median and IQR, to flag comorbidities via Charlson, Elixhauser, PCCC v2, and PCCC v3 and several different utilities in the MIMIC-IV v3.1 dataset. The flagging method, current or cumulative, is reported. Each method was evaluated 10 times.

	medicalcoder::comorbidities()	comorbidity::comorbidity()	MIMIC-IV Code	pccc::ccc()
**charlson**				
current	0.79 (0.76, 0.80)	16.75 (16.63, 17.04)	30.78 (30.60, 30.89)	
cumulative	1.70 (1.60, 1.85)			
**elixhauser**				
current	1.23 (1.17, 1.39)	27.03 (26.92, 27.37)		
cumulative	3.30 (3.21, 3.38)			
**pcccv2.0**				
current	0.92 (0.86, 0.97)			71.60 (71.46, 71.83)
cumulative	2.48 (2.39, 2.61)			
**pcccv3.1**				
current	1.37 (1.29, 1.54)			
cumulative	2.91 (2.85, 2.92)			

10 Summarizing `medicalcoder_comorbidities` Objects

The *medicalcoder* package provides an S3 summary method for the objects returned by `medicalcoder::comorbidities()`. The summaries differ slightly depending on the comorbidity algorithm (`method`). Charlson and Elixhauser return lists with multiple summaries and the PCCC method returns a data.frame of summaries.

When the `flag.method = 'current'`, the `conditions` element of the summary for the Charlson and Elixhauser comorbidities is a data.frame with counts and percentages of the distinct `id.vars` with a given comorbidity, and counts of comorbidities. For PCCC v2 and v3, the return is only this data.frame. When the flag method is cumulative, a warning is given stating that the summary may not be meaningful as it is an encounter level summary.

```
str(summary(medicalcoder_charlson_current), max.level = 1)
## List of 3
## $ conditions :'data.frame': 26 obs. of 4 variables:
## $ age_summary :'data.frame': 1 obs. of 3 variables:
## $ index_summary:'data.frame': 1 obs. of 5 variables:
str(summary(medicalcoder_elixhauser_current), max.level = 1)
## List of 2
## $ conditions :'data.frame': 46 obs. of 3 variables:
## $ index_summary:'data.frame': 2 obs. of 6 variables:
str(summary(medicalcoder_pcccv2.0_current), max.level = 1)
## 'data.frame':    24 obs. of  4 variables:
## $ condition: chr "congeni_genetic" "cvd" "gi" "hemato_immu" ...
## $ label : chr "Other Congenital or Genetic Defect" "Cardiovascular"
```

```
##    "Gastrointestinal" "Hematologic or Immunologic" ...
## $ count : int 9960 159359 56797 35395 85183 211049 17549 39 33640 97078 ...
## $ percent : num 1.45 23.19 8.26 5.15 12.4 ...
str(summary(medicalcoder_pcccv3.1_current), max.level = 1)
## 'data.frame':    24 obs. of  10 variables:
## $ condition : chr "congeni_genetic" "cvd" "gi" "hemato_immu" ...
## $ label : chr "Other Congenital or Genetic Defect" "Cardiovascular"
##    "Gastrointestinal" "Hematologic or Immunologic" ...
## $ dxpr_or_tech_count : int 8156 171247 56613 37617 85384 210879 15114 39 40205
##    97720 ...
## $ dxpr_or_tech_percent : num 1.19 24.92 8.24 5.47 12.42 ...
## $ dxpr_only_count : int 8156 131854 41186 37617 85384 209335 896 39 37338 73916
##    ...
## $ dxpr_only_percent : num 1.19 19.19 5.99 5.47 12.42 ...
## $ tech_only_count : int 0 6159 11327 0 0 244 14210 0 880 4508 ...
## $ tech_only_percent : num 0 0.896 1.648 0 0 ...
## $ dxpr_and_tech_count : int 0 33234 4100 0 0 1300 8 0 1987 19296 ...
## $ dxpr_and_tech_percent: num 0 4.836 0.597 0 0 ...

str(summary(medicalcoder_charlson_cumulative), max.level = 0)
## Warning in .charlson_summary(object): Logic for charlson summary table has been
## implemented for flag.method = 'current'.  Using this function for flag.method =
## 'cumulative' may not provide a meaningful summary.
## List of 3
```

The `data.frame`s are provided so that end users can easily build their own summary tables using tools such as *knitr* Xie (2015) and *kableExtra* (Zhu 2024)

Table 7: Code and resulting table for a summary of the PCCC v2 conditions flagged in the MIMIC-IV dataset.

```r
kableExtra::kbl(
  x = summary(medicalcoder_pcccv2.0_current)[, 2:4],
  format = "latex",
  booktabs = TRUE,
  col.names = c("", "Encounters", "Percent"),
  digits = 2
) |>
kableExtra::kable_styling(
  latex_options = c("striped", "scale_down", "HOLD_position")
) |>
kableExtra::pack_rows("Conditions", start_row =  1, end_row = 13) |>
kableExtra::pack_rows("Total Conditions", start_row = 14, end_row = 24)
```

	Encounters	Percent
**Conditions**		
Other Congenital or Genetic Defect	9960	1.45
Cardiovascular	159359	23.19
Gastrointestinal	56797	8.26
Hematologic or Immunologic	35395	5.15
Malignancy	85183	12.40
Metabolic	211049	30.71
Miscellaneous, Not Elsewhere Classified	17549	2.55
Premature & Neonatal	39	0.01
Neurologic or Neuromuscular	33640	4.90
Renal Urologic	97078	14.13
Respiratory	8272	1.20
Any Technology Dependence	88353	12.86
Any Transplantation	19651	2.86
**Total Conditions**		
Any Condition	371619	54.08
>= 2 conditions	217327	31.62
>= 3 conditions	92827	13.51
>= 4 conditions	26295	3.83
>= 5 conditions	5382	0.78
>= 6 conditions	786	0.11
>= 7 conditions	73	0.01
>= 8 conditions	12	0.00
>= 9 conditions	0	0.00
>= 10 conditions	0	0.00
>= 11 conditions	0	0.00

11 Session Info

Table 8 and Table 9 report details on the machine, R, and the R packages used to produce this document.

Table 8: Details on the machine that generated this document.

Setting	Value
version	R version 4.6.1 (2026-06-24)
os	macOS Tahoe 26.5.2
system	aarch64, darwin23
CPU	Apple M5 Pro
RAM	48.0 GB
date	2026-07-17
pandoc	3.9.0.2 @ /usr/local/bin/ (via rmarkdown)
quarto	1.9.38 @ /usr/local/bin/quarto

Table 9: R packages and versions used to generate this document.

Package	Version	Package	Version	Package	Version	Package	Version
abind	1.4-8	generics	0.1.4	pillar	1.11.1	stringr	1.6.0
backports	1.5.1	ggplot2	4.0.3	pkgconfig	2.0.3	svglite	2.2.2
broom	1.0.13	ggpubr	1.0.0	purrr	1.2.2	systemfonts	1.3.2
car	3.1-5	ggsignif	0.6.4	qwraps2	0.6.3	textshaping	1.0.5
carData	3.0-6	glue	1.8.1	R6	2.6.1	tibble	3.3.1
checkmate	2.3.4	gridExtra	2.3.1	ragg	1.5.2	tidyr	1.3.2
cli	3.6.6	gtable	0.3.6	RColorBrewer	1.1-3	tidyselect	1.2.1
comorbidity	1.1.0	htmltools	0.5.9	Rcpp	1.1.2	tinytex	0.60
cowplot	1.2.0	jsonlite	2.0.0	rlang	1.3.0	vctrs	0.7.3
data.table	1.18.4	kableExtra	1.4.1	rmarkdown	2.31	viridisLite	0.4.3
digest	0.6.39	knitr	1.51	rstatix	1.0.0	withr	3.0.3
dplyr	1.2.1	labeling	0.4.3	rstudioapi	0.19.0	xfun	0.60
evaluate	1.0.5	lifecycle	1.0.5	S7	0.2.2	xml2	1.6.0
farver	2.1.2	magrittr	2.0.5	scales	1.4.0	yaml	2.3.12
fastmap	1.2.0	medicalcoder	0.9.0	sessioninfo	1.2.4		
Formula	1.2-5	otel	0.2.0	stringi	1.8.7		

References

Agency for Healthcare Research and Quality (AHRQ). 2025. *Elixhauser Comorbidity Software Refined for ICD-10-CM Healthcare Cost and Utilization Project (HCUP)*. https://hcup-us.ahrq.gov/toolssoftware/comorbidityicd10/comorbidity_icd10.jsp.

Barrett, Tyson, Matt Dowle, Arun Srinivasan, et al. 2025. *Data.table: Extension of 'Data.frame'*. https://doi.org/10.32614/CRAN.package.data.table.

Beyrer, Julie, Janna Manjelievskaia, Machaon Bonafede, et al. 2021. "Validation of an International Classification of Disease, 10th Revision Coding Adaptation for the Charlson Comorbidity Index in United States Healthcare Claims Data." *Pharmacoepidemiology and Drug Safety* 30 (5): 582–93.

Complex Chronic Conditions Version 3.0. 2024. Children's Hospital Association; https://www.childrenshospitals.org/content/analytics/toolkit/complex-chronic-conditions.

DeWitt, Peter, James Feinstein, and Seth Russell. 2026. *Pccc: Pediatric Complex Chronic Conditions*. https://github.com/CUD2V/pccc.

Feinstein, James A, Matt Hall, Amber Davidson, and Chris Feudtner. 2024. "Pediatric Complex Chronic Condition System Version 3." *JAMA Network Open* 7 (7): e2420579–79. https://doi.org/10.1001/jamanetworkopen.2024.20579.

Feinstein, James A., Seth Russell, Peter E. DeWitt, Chris Feudtner, Dingwei Dai, and Tellen D. Bennett. 2018. "R Package for Pediatric Complex Chronic Condition Classification." *JAMA Pediatrics* 172 (6): 596–98. https://doi.org/10.1001/jamapediatrics.2018.0256.

Feudtner, Chris, James A Feinstein, Wenjun Zhong, Matt Hall, and Dingwei Dai. 2014. "Pediatric Complex Chronic Conditions Classification System Version 2: Updated for ICD-10 and Complex Medical Technology Dependence and Transplantation." *BMC Pediatrics* 14: 1–7. https://doi.org/10.1186/1471-2431-14-199.

Gasparini, Alessandro. 2018. "Comorbidity: An r Package for Computing Comorbidity Scores." *Journal of Open Source Software* 3: 648. https://doi.org/10.21105/joss.00648.

Glasheen, William P, Tristan Cordier, Rajiv Gumpina, Gil Haugh, Jared Davis, and Andrew Renda. 2019. "Charlson Comorbidity Index: ICD-9 Update and ICD-10 Translation." *American Health & Drug Benefits* 12 (4): 188. https://pubmed.ncbi.nlm.nih.gov/31428236/.

Goldberger, Ary L., Luis A. N. Amaral, Leon Glass, et al. 2000. "PhysioBank, PhysioToolkit, and PhysioNet: Components of a New Research Resource for Complex Physiologic Signals."

Circulation 101 (23): e215–20. https://doi.org/10.1161/01.cir.101.23.e215.

Johnson, Alistair E W, David J Stone, Leo A Celi, and Tom J Pollard. 2018. "The MIMIC Code Repository: Enabling Reproducibility in Critical Care Research." *Journal of the American Medical Informatics Association* 25 (1): 32–39.

Johnson, Alistair EW, Lucas Bulgarelli, Lu Shen, et al. 2023. "MIMIC-IV, a Freely Accessible Electronic Health Record Dataset." *Scientific Data* 10 (1): 1.

Johnson, Alistair, Lucas Bulgarelli, Tom Pollard, et al. 2024. "MIMIC-IV." *PhysioNet*, ahead of print, October. https://doi.org/10.13026/kpb9-mt58.

Ludvigsson, Jonas F, Peter Appelros, Johan Askling, et al. 2021. "Adaptation of the Charlson Comorbidity Index for Register-Based Research in Sweden." *Clinical Epidemiology*, 21–41. https://doi.org/https://doi.org/10.2147/CLEP.S282475.

Ludvigsson, Jonas F, Peter Appelros, Johan Askling, et al. 2023. "Adaptation of the Charlson Comorbidity Index for Register-Based Research in Sweden [Corrigendum]." *Clinical Epidemiology* 15: 753–54. https://doi.org/https://doi.org/10.2147/clep.s425901.

Mersmann, Olaf. 2024. *Microbenchmark: Accurate Timing Functions*. https://doi.org/10.32614/CRAN.package.microbenchmark.

Quan, Hude, Bo Li, Colette M. Couris, et al. 2011. "Updating and Validating the Charlson Comorbidity Index and Score for Risk Adjustment in Hospital Discharge Abstracts Using Data from 6 Countries." *American Journal of Epidemiology* 173 (6): 676–82. https://doi.org/10.1093/aje/kwq433.

Quan, Hude, Vijaya Sundararajan, Patricia Halfon, et al. 2005. "Coding Algorithms for Defining Comorbidities in ICD-9-CM and ICD-10 Administrative Data." *Medical Care* 43 (11): 1130–39. https://doi.org/10.1097/01.mlr.0000182534.19832.83.

Sundararajan, Vijaya, Toni Henderson, Catherine Perry, Amanda Muggivan, Hude Quan, and William A Ghali. 2004. "New ICD-10 Version of the Charlson Comorbidity Index Predicted in-Hospital Mortality." *Journal of Clinical Epidemiology* 57 (12): 1288–94.

Xie, Yihui. 2014. "Knitr: A Comprehensive Tool for Reproducible Research in R." In *Implementing Reproducible Computational Research*, edited by Victoria Stodden, Friedrich Leisch, and Roger D. Peng. Chapman; Hall/CRC.

Xie, Yihui. 2015. *Dynamic Documents with R and Knitr*. 2nd ed. Chapman; Hall/CRC. https://yihui.org/knitr/.

Xie, Yihui. 2025. *Knitr: A General-Purpose Package for Dynamic Report Generation in R*. https://yihui.org/knitr/.

Zhu, Hao. 2024. *kableExtra: Construct Complex Table with 'Kable' and Pipe Syntax*. https://doi.org/10.32614/CRAN.package.kableExtra.
